# The geometry of admixture in population genetics: the blessing of dimensionality

**DOI:** 10.1093/genetics/iyae134

**Published:** 2024-08-14

**Authors:** José-Angel Oteo, Gonzalo Oteo-García

**Affiliations:** Departament de Física Teòrica, Universitat de València, Burjassot, Valencia 46100, Spain; Institute for Integrative Systems Biology, Paterna, Valencia 46980, Spain; Dipartamento di Biologia Ambientale, Sapienza Università di Roma, Rome 00185, Italy; Centre for Palaeogenetics and Department of Archaeology and Classical Studies, Stockholm University, Stockholm SE-106 91, Sweden

**Keywords:** population admixture, *f*-statistics, genetic drift, dimensional reduction

## Abstract

We present a geometry-based interpretation of the *f*-statistics framework, commonly used in population genetics to estimate phylogenetic relationships from genomic data. The focus is on the determination of the mixing coefficients in population admixture events subject to post-admixture drift. The interpretation takes advantage of the high dimension of the dataset and analyzes the problem as a dimensional reduction issue. We show that it is possible to think of the *f*-statistics technique as an implicit transformation of the genomic data from a phase space into a subspace where the mapped data structure is more similar to the ancestral admixture configuration. The 2-way mixing coefficient is, as a matter of fact, carried out implicitly in this subspace. In addition, we propose the admixture test to be evaluated in the subspace because the comparison with the conventional one provides an important assessment of the admixture model. The overarching geometric framework provides slightly more general formulas than the *f*-formalism by using a different rationale as a starting point. Explicitly addressed are 2- and 3-way admixtures. The mixture proportions are provided by suitable linear fits, in 2 or 3 dimensions, that can be easily visualized. The difficulties encountered with introgression and gene flow are also addressed. The developments and findings are illustrated with numerical simulations and real-world cases.

## Introduction

The determination of admixture proportions in hybrid populations is a central topic in population genetics. At large evolutionary time scales, a population admixture event can be thought of as a sudden process in which a new linage emerges as the genetic weighted combination of 2 or more donors, in its simplest formulation.

The formalism known as *f*-statistics (Patterson *et al*. 2012; Haak *et al*. 2015; Lazaridis *et al*. 2016; Peter 2016, 2022; Lipson 2020; Harney *et al*. 2021), which deals with allele frequency correlations between 2, 3, and 4 populations, is a commonly used technique to determine the admixture coefficients. Despite their simple computation and definition, the *f*-statistics outcome assessment may not be straightforward (Lipson 2020). We develop a geometry-based interpretation of *f*-statistics intended to deepen the understanding of the population admixture problem. Geometric-like methods have already been raised in the past (Cavalli-Sforza 1966; Cavalli-Sforza and Piazza 1975; Long 1991; Cavalli-Sforza *et al*. 1994; Brisbin *et al*. 2012; Oteo-García 2020; Agranat-Tamir *et al*. 2021). The strength of our approach is to take advantage of the high dimensionality of the problem, broadening the *f*-formalism toolkit.

Whenever the genetic information from the original populations is available, determining the admixture proportions is straightforward. However, the evolutionary history of the original populations manifests itself, for example, in random changes in allele frequencies, a phenomenon known as genetic drift. Although ancient DNA can sometimes aid in estimating admixture proportions by providing better proxies, the time elapsed since admixture and the possibility that the putative parents themselves are extinct make accurate estimates difficult. Moreover, over time, a population may experience continuous, discrete, occasional, or repeated genetic influx from other populations, which makes the issue more challenging. The present study is concerned with the approximation of admixture proportions in populations that following the hybridization are assumed to have evolved in time solely via genetic drift. A population level dataset with allele frequencies corresponding to a large number of Single-Nucleotide Polymorphisms (SNPs) is a common starting point for dealing with these questions.

**Fig. 1.**
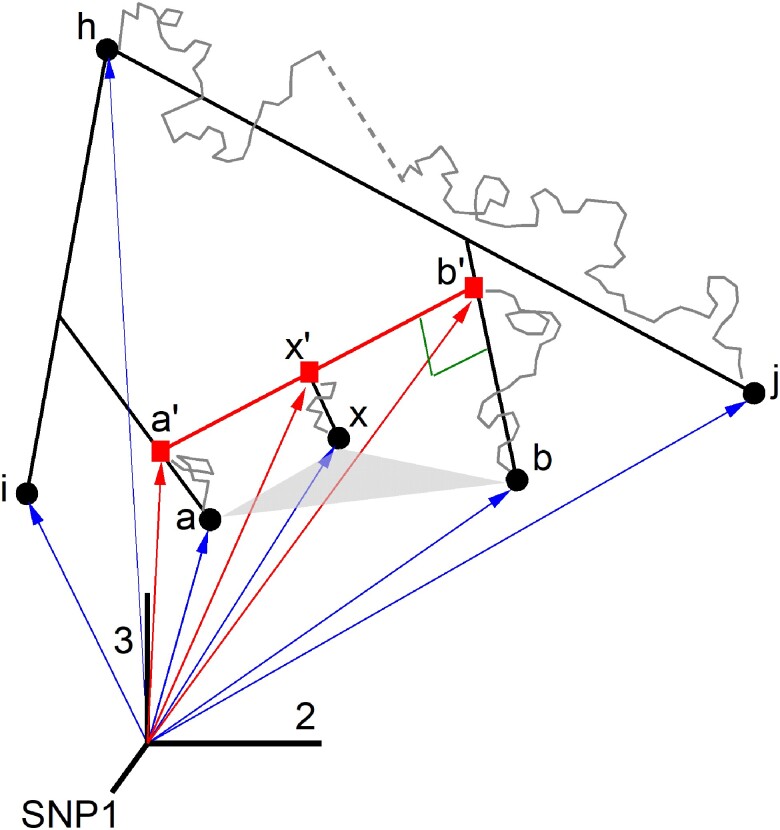
Three-dimensional allele frequency space. Populations $a^{\prime },b^{\prime }$ are the donors and $x^{\prime }$ the hybrid at admixture inception. They evolve to a,x,b (proxies in the dataset) via genetic drift (Brownian-like gray curves). The Brownian-like trajectory from *h* to *j* is broken up by a random jump, which is intended to represent an event such as a population bottleneck. The triangle △axb is useful in the discussion because the geometric condition for *x* to be a hybrid of *a* and *b* is that the angle at vertex *x* be obtuse.

The population admixture problem may be posed as a geometric issue in a phase space, the allele frequency space, where each population is represented by a point and each axis corresponds to an SNP in the dataset (Oteo-García and Oteo 2021). With only 3 SNPs, Fig. 1 outlines the nature of the problem. Populations $i,a^{\prime },b^{\prime },a,b,j$, are assumed to be related by phylogenetic treeness and are located in phase space by their position vectors whose coordinates are given by the allele frequencies in the dataset. At the time of admixture, we have 3 co-linear points, $a^{\prime },x^{\prime },b^{\prime }$, with $a^{\prime }$ and $b^{\prime }$ the donors and $x^{\prime }$ the hybrid. Then, they describe Brownian-like trajectories because allele frequencies undergo small random changes as a result of genetic drift, resulting in population proxies a,x,b.

Populations *i* and *j*, which appear as spectators in Fig. 1, will be referred to as auxiliary populations and will play an important role. Any population related to the admixture contributors phylogenetically may be considered as an auxiliary. At the early stages of *f*-statistics, these auxiliary populations were coined *outgroup* populations and currently referred to as *right* or *reference* populations.

In Fig. 1, population *h* stands for the common ancestor of populations *i* and *j*. The Brownian-like trajectory depicted from *h* to *j* is interrupted by a *random jump*, which is meant to represent a sudden event that may occur in long evolutionary histories.

The geometric condition for *x* to be a hybrid is $90^{\circ }<\phi <180^{\circ }$, where *ϕ* is the angle at vertex *x* in triangle △axb. Otherwise, whenever the 3 angles are acute, the populations a,x,b, are mathematically a phylogenetic tree (Oteo-García and Oteo 2021). A difficulty associated with long evolutionary histories is that an admixture triangle in phase space may evolve to have 3 acute angles, fooling the test $90^{\circ }<\phi <180^{\circ }$. And conversely, a phylogenetic scenario may evolve to appear mathematically as admixture (Cavalli-Sforza *et al*. 1994). This is why complementary knowledge about the population history is important.

The 2-way population admixture issue that we are addressing here is stated as follows. Determine the relative length of the segment $\bar{x^{\prime }b^{\prime }}$ with respect to $\bar{a^{\prime }b^{\prime }}$, when we only know the proxy triangle △axb. The solution provides an estimate of the admixture proportions. No reconstruction of the ancestral allele frequencies is contemplated. The 3-way scenario may be also posed in these terms, albeit it requires a more elaborate formulation.

A crucial ingredient in the approach is the high dimension of the allele frequency space, fixed by the number of SNPs sequenced, typically in the hundreds of thousands. Geometry in high dimensions presents challenges because the space is *too empty*. The conventional example to illustrate it considers the volume of a cube of side 2 in $R^{n}$, which is $2^{n}$ bigger than the volume of a unit cube, despite the fact that their sides are only a factor 2 apart. *Curse of dimensionality* is the term used to characterize this feature. Fortunate enough, there are also positive aspects associated with high dimensionality sometimes referred to as the *blessing of dimensionality*. In this sense, we will use (i) the Johnson–Lindenstrauss (JL) lemma about dimension reduction, which states that pairwise distances are approximately preserved after projection onto a random subspace; (ii) the Brownian-like character (induced by genetic drift) of the population time trajectories in phase space, and (iii) the quasi-orthogonality of random vectors in high dimension.

Equipped with these 3 elements, we will show how the post-admixture drift is depleted when computing in a random subspace defined by a number of auxiliary populations. Also, how the current *f*-statistics estimate of the 2-way mixing coefficient may be understood as a computation in such subspace. Moreover, we will explain the advantage of computing the conventional population admixture test in the random subspace and will provide an explicit formula to this end.

Section presents the description of the overarching geometric framework and its relationship with the standard *f*-statistics formalism. Section gathers a number of numerical simulations based on real-world data intended to illustrate every aspect described in the preceding Section. Section contains final comments and discussion of results. The Supplementary document gathers the mathematical details as well as an application to real data.

## Population admixture, geometry, and *f*-statistics

The geometric interpretation of the *f*-statistics framework is explained in this Section. Different aspects of the population admixture problem are progressively introduced. The emphasis is on the geometric formalism description of the admixture problem, the admixture proportion determination and the assessment of the model. We commence with the 2-way problem and then generalize to 3-way admixture. Only essential formulas are kept in the main text. Formal developments and additional details are deferred to the Supplementary document which is intended to be self-contained. In particular, we deal with the difficulties associated with gene flow and introgression. From an instrumental point of view, the mathematical developments rely solely on the definition and the geometric interpretation of the scalar product of 2 vectors. All the results are expressed in terms of the 3 *f*-statistics, $f_{2}$, $f_{3}$, and $f_{4}$ whose definitions and phylogenetic interpretations are in Supplementary Section S.1.

### The recovery of the ancestral 2-way population admixture state

**Fig. 2.**
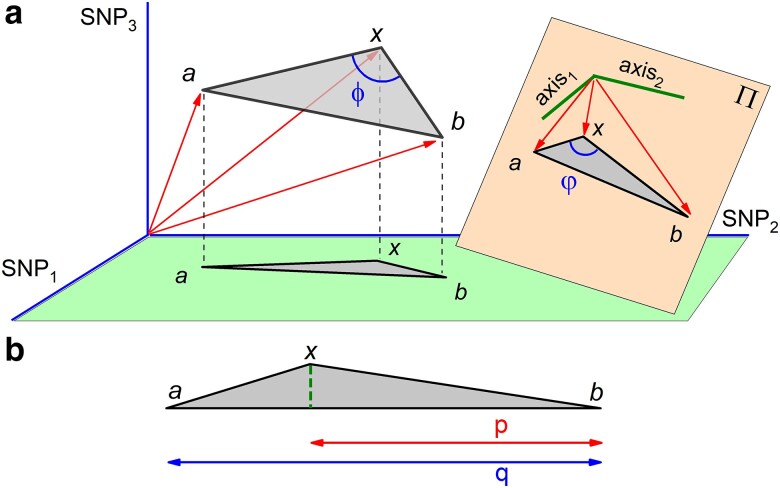
(a) The population admixture scenario with 3 SNPs and the projection on the random plane *Π*. (b) Determination of the admixture proportion as p/q.

Suppose we have a dataset with the allele frequencies of 3 SNPs of six populations. Three of these populations correspond to the proxies of a 2-way admixture scenario. The remaining 3 are *auxiliary* populations. We can visualize the dataset in a 3D phase space with SNPs as axes and populations as points whose coordinates are allele frequencies (see Figs. 1 and 2a). The vectors of the admixture population proxies a,b, and the hybrid *x*, define a triangle. Nonetheless, at the time of mixture the 3 points were aligned in space, as mentioned above. The angle *ϕ* at vertex *x* (see Fig. 2a) must be obtuse if the population admixture test is to be passed. It is the geometric interpretation of the current admixture test, $f_{3}(a,b;x)<0$.

By pairing the remaining 3 points, the auxiliary populations, we define 2 arbitrary directions (axis1 and axis2) that, in turn, define the random plane *Π* that contains them (see Fig. 2a). The projection of the triangle onto *Π*, or even onto the horizontal plane, shows that the relative pairwise distances between vertices get distorted. Moreover, we could reorient the plane *Π* (using new auxiliary populations) to produce infinitely many edge patterns, running from the very same triangle to a pure segment. Paradoxically, in high dimension the distortion of such a random projection is negligible, according to a mathematical result due to JL (Johnson *et al*. 1986). Projecting the triangle of a dataset with a large number of SNPs onto a random subspace generated by a few auxiliary populations (the JL subspace) provides an undistorted triangle (approximately).

This projection from the full allele frequency space of high dimension onto the low dimension JL subspace defined by auxiliary populations would be a mere mathematical curiosity were it not for an additional high dimension effect we explain next.

The vertices of the triangle, which were aligned at the time of admixture, are subject to the random effect of genetic drift and continuously driven to a different place in a Brownian-like trajectory. The crucial point is that the projection onto the JL subspace has the collateral effect of depleting the genetic drift displacements. This occurs because random vectors in high dimension, which is the way we treat genetic drift, are quasi-orthogonal to any arbitrary direction with great probability (Vershynin 2018). Thus, the projected triangle is a combination between preservation of edge relative proportions and suppression of genetic drift. An effect of this kind has been noted in the *f*-formalism, from statistical grounds. The $f_{4}$ values computed with proxies should be similar to those computed with original populations (Patterson *et al*. 2012).

The geometric viewpoint goes beyond and allows to estimate the angle φ at vertex *x* in the projected, or *post-JL*, triangle. In Fig. 2a, φ is depicted on the plane *Π*. A value $\varphi ∼180^{\circ }$ witnesses the success of the post-JL reconstruction of the admixture model. This estimate is by far more reliable than the current test for 2-way population admixture, $f_{3}<0$; or $90^{\circ }<\phi <180^{\circ }$. The reason is that the *ϕ* measurements are carried out in the full phase space and therefore are more flawed by post-admixture drift, a fact contributing to fool the test. This is the novel contribution of the geometric formalism.

To be more specific, the 2-way mixture coefficient is obtained from a simple computation based on the triangle edges in phase space with the population admixture triplet at vertices. Regardless whether or not in the full phase space or in the JL subspace, the coefficient is simply the ratio p/q in Fig. 2b. For a well-posed population admixture model, the projected triangle onto the JL subspace is flatter than the one in the full phase space. Therefore, the post-JL reconstruction of the admixture triangle provides much more accurate segments *p* and *q*.

The golden rule is to make the estimates of both the admixture test, or angle, and the mixture coefficient in a random subspace defined by auxiliary populations and not in the full phase space of allele frequencies.

In practice, the mixing coefficient can be obtained as the slope of a linear fit through zero of $f_{4}$ quantities and explicit formulas are provided for the admixture angles. This procedure allows to visualize why pairs of auxiliary populations with large shared drift with donors are very relevant.

The geometric picture allows us to understand why in an introgression regime the approximate depleting of the post-admixture drift is a delicate issue.

### Practical results: 2-way admixture

The ideas concerning the 2-way population admixture problem take the following form. The starting point is the relationship between donors and hybrid, approximately described by the linear combination of vectors


(1) $${\vec{\;p}}_{x}=\alpha {\vec{\;p}}_{a}+(1-\alpha ){\vec{p}}_{b},\;\alpha \in [0,1],$$

in the allele frequency space. The components of a vector ${\vec{p}}_{a}$ are the *s* allele frequencies $\left\{p_{a}^{k}\right\}$, k=1,…,s, of population *a* in the dataset and *α* is the mixing coefficient. The fact that there is just one parameter, *α*, is what makes the 3 populations *a*, *x*, and *b* to stay on a straight line at the inception of the admixture. The allele frequency vectors, which contain the dataset information, are the inputs and we want the value of the mixing proportion *α*. Viewed as an algebraic linear system of *s* equations and just one unknown, *α*, the hope is to find an estimate in the sense of Least Squares (LS). The solution can be presented in terms of the 3 *f*-statistics (see Supplementary Section S.2). This is because the *f*-statistics can be readily expressed in terms of the scalar (or dot) product of 2 vectors (see Supplementary Section S.1), which is the algebraic tool used in the geometric framework (Oteo-García and Oteo 2021).

The LS estimate in full allele frequency space reads


(2) $$\alpha =\frac{\;f_{3}(a,x;b)}{f_{2}(a,b)},$$

for the mixing proportion. In practice, one can equivalently use a numerical routine to fit the set $\left\{p_{a}^{k}-p_{b}^{k},p_{x}^{k}-p_{b}^{k}\right\}$ (from (1)) with a straight line through the origin and obtain the slope (*α*) and its confidence interval.

The admixture angle is


(3) $$\cos\;\phi =\frac{\;f_{3}(a,b;x)}{[f_{2}(a,x)f_{2}(b,x){]}^{1/2}}.$$

As mentioned above, the admixture test reads


(4) $$90^{\circ }<\phi <180^{\circ }\;\mathrm{or}\;f_{3}(a,b;x)<0.$$

Notice that, unlike *ϕ*, $f_{3}$ has not a bounded scale.

In the JL subspace spawned by *k* auxiliary populations i,j, which has dimension k(k−1)/2, the system (1) reads


(5) $$f_{4}^{\prime }(x,b;i,j)=\alpha f_{4}^{\prime }(a,b;i,j),\;i<j=1,\ldots ,k,$$

where the prime stands for an $f_{4}$ statistic renormalized as $f_{4}^{\prime }(a,b;i,j)=\sqrt{s}\;f_{4}(a,b;i,j)/\sqrt{\;f_{2}(i,j)}$, that emerges naturally in the projection process onto the JL subspace. The corresponding LS solution reads


(6) $$\alpha =\frac{\sum_{i<j}^{k}f_{4}^{\prime }(x,b;i,j)f_{4}^{\prime }(a,b;i,j)}{\sum_{i<j}^{k}f_{4}^{\prime 2}(a,b;i,j)}.$$

From the practitioner perspective, an equivalent alternative to computing (6) is to generate the 2D plot with the points $\left\{f_{4}^{\prime }(a,b;i,j),f_{4}^{\prime }(x,b;i,j)\right\}$, i<j=1,…,k; and use a standard routine to fit a straight line through zero to get the slope *α* and its confidence interval. An instance of this kind can be found in (Haak *et al*. 2015, Supplementary information).

For the projected admixture angle, we get


(7) $$\cos\;\varphi =\frac{\sum_{i<j}^{k}f_{4}^{\prime }(x,a;i,j)f_{4}^{\prime }(x,b;i,j)}{[\sum_{i<j}^{k}f_{4}^{\prime 2}(x,a;i,j)\sum_{i<j}^{k}f_{4}^{\prime 2}(x,b;i,j){]}^{1/2}}.$$

The closer the angle φ to $180^{\circ }$, the better the post-JL reconstruction of the admixture state.

### Remarks about the estimation of *α*

The *α* determination in (2) is accurate for short enough evolutionary times so as the post-admixture drift effect is small. It can be also useful whenever the full phase space in which the dataset is embedded is of low dimension. Such an instance is the space of self-consistent ancestry states generated by the ADMIXTURE code (Alexander *et al*. 2009; Oteo-García 2020; Agranat-Tamir *et al*. 2021).

For the discussion of the *α* estimate provided by (6), it is convenient to remind that $f_{4}(a,b;i,j)$ is a measure of the shared genetic drift between population pairs (a,b) and (i,j) in a phylogenetic tree.

If we think of a situation with only 2 auxiliary populations (k=2), then (6) collapses to


(8) $$\alpha =\frac{\;f_{4}(x,b;1,2)}{f_{4}(a,b;1,2)}.$$

This is the so-called $f_{4}$-ratio index (Patterson *et al*. 2012) defined at the early stage of the *f*-statistics. The geometric perspective tells us that it corresponds to a drastic projection onto a subspace of dimension one and the JL lemma foresees a large distortion in this case. This situation is illustrated at depth in Supplementary Section S.5. A possible remedy would be to compute a number of $f_{4}$-ratios, with different auxiliary pairs, followed by a statistical treatment leading to a realistic estimate of *α*. This is what implicitly happens in (6), as we show next.

Equation (6) corresponds to the formula that gives the slope of a linear fit through the origin to the set of points $\left\{f_{4}^{\prime }(a,b;i,j),f_{4}^{\prime }(x,b;i,j)\right\}$, with i<j=1,…,k. Now, a straight line fit through the origin can be interpreted as a weighted average of the slopes defined by every single point in the plot, where the weight is the horizontal squared distance of that point to the origin. In the fit y=mx to a set of *n* points, the slope reads $m=\sum_{i}y_{i}x_{i}/\sum_{i}x_{i}^{2}=\sum_{i}m_{i}\;x_{i}^{2}/\sum_{i}x_{i}^{2}$, where $m_{i}=y_{i}/x_{i}$ is the slope defined by the point $\left(x_{i},y_{i}\right)$, and i=1,…,n. Thus $x_{i}^{2}$ are weights. Following that trick, equation (6) is the slope *m*, obtained as the average of a number n=k(k−1)/2 of $f_{4}$-ratios, namely $y=\;f_{4}(x,b;i,j)/\;f_{4}(a,b;i,j)$, and weighted by the renormalized squared shared drift between the donor and the auxiliary pairs, i.e. $x=f_{4}^{\prime 2}(a,b;i,j)$. We are led to conclude that, as a rule, auxiliary population pairs with large shared drift (large *x*) with donors are the relevant ones in the estimation of the ancestral mixing proportion because they own the largest weights in the average. Auxiliary pairs with negligible $x=f_{4}^{\prime 2}(a,b;i,j)$ yield points that appear close to the origin in the regression plot and hardly contribute to the estimate of the slope *m* of the fit.

Less clear is the situation if auxiliary pairs with not so negligible shared drift do enter the game in a large number. It may give rise to a large diversity of single $f_{4}$-ratio values whose collective contribution spoils the weighted average. This drawback could be connected with a recent observation in a thorough simulation-based study (Harney *et al*. 2021) which warns, under statistical grounds, against using a large number of auxiliary populations (right or reference populations in Harney *et al*. 2021) because it spoils the *P*-values of the admixture analyses leading to contradictory conclusions. The critical number is not disclosed but seems to depend on both the population history and the total amount of data. Certainly, this seems to contradict the JL lemma in the sense that the larger the subspace dimension (i.e. larger number of auxiliary populations) the less distortion after the JL projection. The geometric framework may explain it as a cumulative excess of points moderately close to the origin in the 2D plot, providing a large diversity of single slopes (namely, $f_{4}$-ratios) that eventually affects the average values of the analysis. The 2D visualization of the data would help efficiently to detect this phenomenon and other unexpected artifacts as the one described in Supplementary Section S.9.

**Fig. 3.**
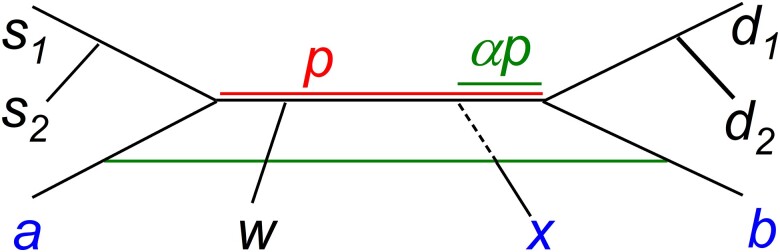
Phylogenetic graph. Populations *a* and *b* are the 2-way donors and *x* the hybrid, where the *a* contributing proportion is *α*. Tips $s_{1},s_{2},w,d_{1},d_{2}$ are auxiliary populations and *p* stands for the shared drift between pairs (a,b) and ($s_{i},d_{j}$). The lower horizontal line is meant to stand for the admixture.

These cases described may be interpreted in connection with the phylogenetic graph of Fig. 3. Auxiliary pairs with an element from $s_{i}$ and one from $d_{i}$, namely $\left\{s_{i},d_{j}\right\}$, have a large shared drift with donors, whereas auxiliary pairs $\left\{s_{1},s_{2}\right\}$ and $\left\{d_{1},d_{2}\right\}$ do not. The former ones will really compute the *α* estimate, each one weighted by the Euclidean distances $f_{2}$ in the $f_{4}^{\prime }$ terms. Auxiliary pairs $\left\{s_{i},w\right\}$ and $\left\{w,d_{i}\right\}$ present the intermediate situation. In the 2D regression plot, they locate at intermediate distances.

There is a tendency among practitioners to consider the here introduced auxiliary populations as distantly related to the donors. This may be rooted (Haak *et al*. 2015) in the fact that in the early stages of the *f*-formalism, what we have termed auxiliary populations were treated and referred to as outgroups. In our discussion, we emphasize that the key point is not genetic distance but shared drift.

### Three-way population admixture

The JL projection procedure may be generalized to a population admixture with more than 2 donors. Next, we illustrate the 3-way case with contributors a,b,c.

In the case when the 3 donors are phylogenetically related, a necessary mathematical condition for *x* to be a hybrid is that the 4 populations {a,b,c,x} be not a phylogenetic tree, namely


(9) $$f_{4}(x,a;b,c)\neq 0,\;f_{4}(x,c;a,b)\neq 0,\;f_{4}(x,b;c,d)\neq 0,$$

where we have used Supplementary equation (S.4). Under the hypothesis that all the *s* allele frequencies are combined with fixed proportions, the linear combination now reads


(10) $${\vec{p}}_{x}=\alpha {\vec{p}}_{a}+\beta {\vec{p}}_{b}+(1-\alpha -\beta ){\vec{p}}_{c},\;\alpha ,\beta \in [0,1].$$

**Fig. 4.**
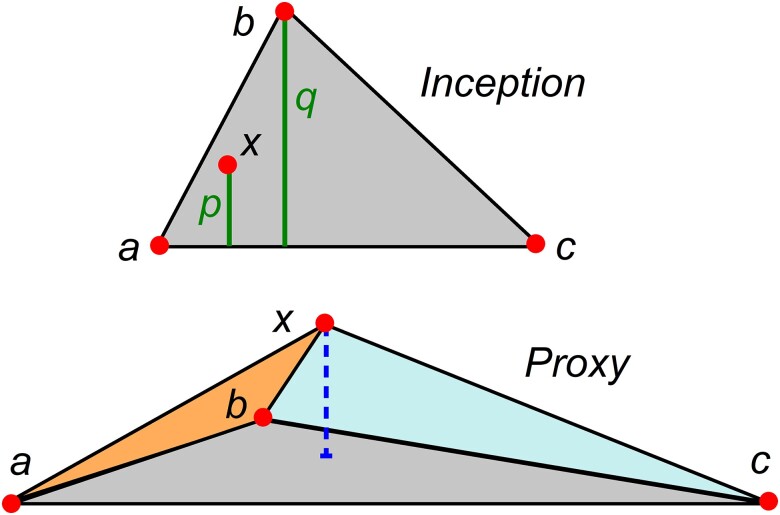
Upper panel: 3-way admixture at inception in allele frequency space. p/q is the contributing fraction of donor *b*. Bottom panel: post-admixture effect on the 3-way admixture.

The 3-way population admixture in the allele frequency space appears, at its inception, as a triangle with donors at vertices and the hybrid as an inner point. An explicit proof may be found in Supplementary Section S.16. In the example of Fig. 4 (upper panel), the donor *c* appears as the least contributor. The acute angles at the 3 vertices witness that the donors are phylogenetically related. In the course of time, genetic drift shifts the 4 points to form a tetrahedral structure (bottom panel).

The basic approach to estimate *α* and *β*, and their confidence intervals, goes through a numerical fit in the allele frequency space to the set $\left\{p_{a}^{k}-p_{c}^{k},p_{b}^{k}-p_{c}^{k},p_{x}^{k}-p_{c}^{k}\right\}$, with a plane through the origin. The limitations are the same as in 2-way admixture: this determination is good for shorter evolutionary times.

After JL projection with d=k(k−1)/2 pairs of auxiliary populations the system (10) reads


(11) $$f_{4}^{\prime }(x,c;i,j)=\alpha f_{4}^{\prime }(a,c;i,j)+\beta f_{4}^{\prime }(b,c;i,j),\;i<j=1,\ldots ,k.$$

A way to look at the LS solution of the system consists in finding the best fit in 3D to a plane through the origin with the *d* points in the set $\left\{f_{4}^{\prime }(a,c;i,j\right)$, $f_{4}^{\prime }(b,c;i,j)$, $f_{4}^{\prime }(x,c;i,j)\}$. The explicit LS solution is in Supplementary Section S.12. As in the case of 2-way admixture, we could equivalently use a numerical routine to fit a plane through the origin to the set of points and get the parameter estimates and their confidence intervals.

The formal geometric interpretation of the mixing coefficients in terms of tetrahedron edge projections is the same whether in the full phase space or the JL subspace, but is not easy to visualize. It is simple in the particular case of population admixture at its inception. For instance, the contributing fraction of donor *b* is given by the ratio p/q illustrated in the upper panel of Fig. 4.

The alignment of the 3 populations of a 2-way admixture is measured in the JL subspace by the angle φ. It would be convenient to have an index to measure to what extent the post-admixture tetrahedron gets flattened in the JL subspace and therefore similar to an admixture at its inception. In order to have a dimensionless quantity, we have defined a heuristic *flatness* index as the ratio of the tetrahedron height over the square root of its base area: $h/\sqrt{A}$. We have computed it in terms of the tetrahedron volume *V* and the base area *A* in both the full phase space and the JL subspace as $3V/A^{3/2}$ (see Supplementary Section S.16). *V* and *A* are computed in terms of $f_{2}$’s.

When the tetrahedron is not warped enough for the orthogonal projection of the *x* vertex to fall outside the base, this indicator should be useful. Once more, the introgression regime is more sensitive to this circumstance.

In Supplementary Section S.14, a variant of the problem is treated in which the admixture takes place in 2 phases via a transitional hybrid. As an extension of this scenario, in Supplementary Section S.15 a modest model of gene flow is posed.

## Numerical simulations

We have run a number of simulations using real-world data to illustrate the formal arguments presented above. To this end, we have used the population dataset *v37.2.1240K_HumanOrigins* from Reich Lab (Mallick and Reich 2023) to build up a dataset of 40 populations: English, Yoruba, Biaka, Mbuti, French, Druze, Sardinian, Italian_North, Basque, Adygei, Nganasan, Lithuanian, Lebanese, Han, Papuan, Surui, Karitiana, Aleut, Ju_hoan_North, Gujarati, Iranian, Australian, Khomani, Yukagir, Chukchi, Ulchi, Tubalar, Koryak, Even, Brahui, Balochi, Hazara, Makrani, Pathan, Kalash, Burusho, Japanese, Orcadian, Mayan, and Russian. The effective number of SNPs is s=597.568.

We have built up hybrid populations as in the linear combinations (1) and (10) taking 2, or 3, populations from the set whereas the remaining populations in the dataset serve as auxiliary populations. To simulate post-admixture drift, noise of amplitude ϵ∈[0,0.5] has been added to the allele frequencies of the donors and the hybrid. The quantity added is ϵδ, with δ∈[−1,1] a random number. If the resulting frequency is out of range a new *δ* is generated. This type of simulation takes only account of genetic drift, which is the assumption referred to in the Introduction. The idea is to buttress the preceding formal developments with visual evidence that gives the reader confidence in the geometrical interpretation. This is important if one is to rely on the usefulness of the post-JL angle, φ. For more complex simulations, e.g. controlling the mutation and recombination rates, and the effective population sizes, special purpose codes like MSPRIME (Kelleher *et al*. 2016; Baumdicker *et al*. 2022) should be used.

### Results for 2-way admixture

**Fig. 5.**
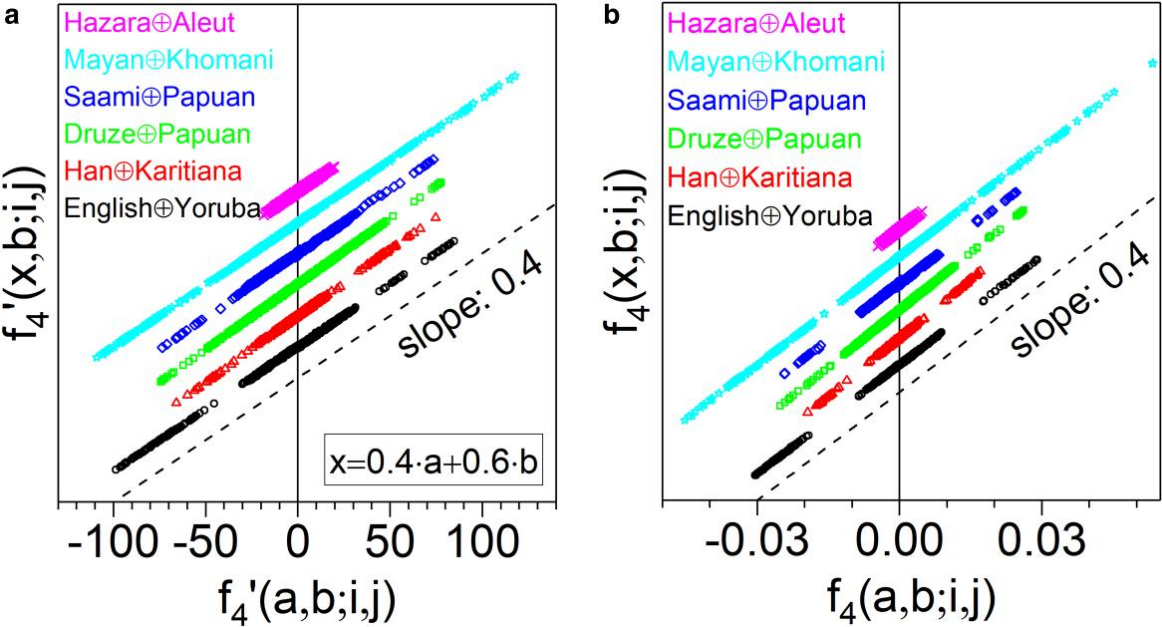
$f_{4}^{\prime }$ and $f_{4}$ representations of the six different 2-way admixture simulations given in the legend, following the model x=0.4a+0.6b. Every data alignment in the plot corresponds to one hybrid, contains 703 points (auxiliary population pairs i,j) and has been vertically shifted for readability. The dashed line has slope: 0.4, and is given for visual reference. The legend is ordered as the alignments sequence. a) Renormalized, and b) Standard

We commence with a number of admixture simulations, ${\vec{\;p}}_{x}=0.4\;{\vec{\;p}}_{a}+0.6\;{\vec{\;p}}_{b}$, shown in Fig. 5 with the donors specified in the legend. The dotted line has slope 0.4 and is given for visual reference. Notice that both representations $f_{4}^{\prime }$ and $f_{4}$ are good linear point distributions. The different admixture models have been vertically shifted for readability. This is the first evidence that the law in (5) makes sense, as it does the corresponding unprimed equation. Notice that although the set of auxiliary populations is very similar in all simulations, the plots exhibit a variety of scale ranges as a consequence of the diversity in the shared drift between the auxiliary pairs and the contributors.

**Fig. 6.**
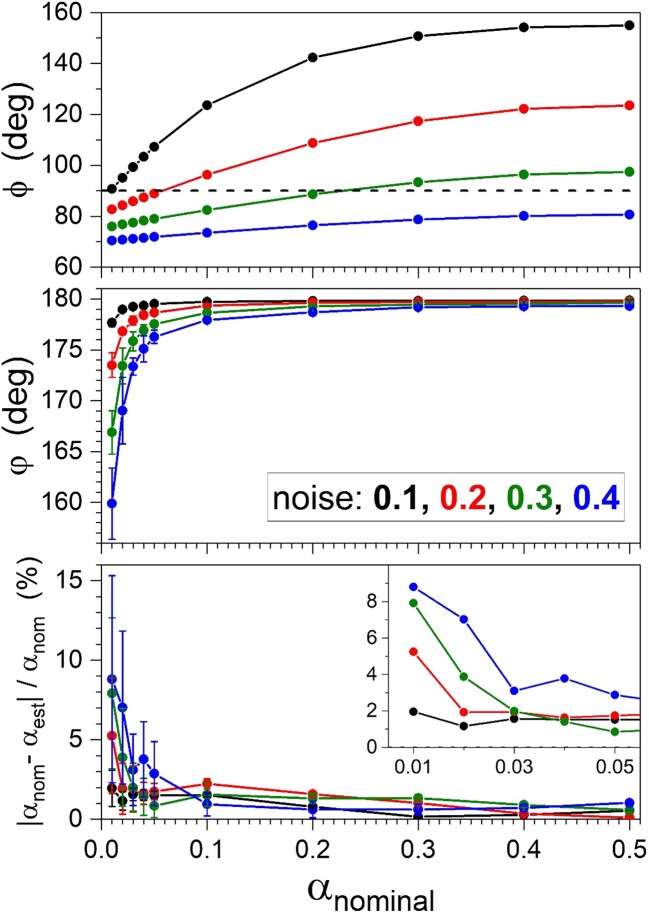
Admixture model: α⋅English +  (1−α)⋅Yoruba. Results for the angles φ and *ϕ* and the relative absolute error of the *α* estimate, as a function of the nominal *α*, for several noise levels. The inset zooms the introgression regime.

For the particular model α⋅English +  (1−α)⋅Yoruba, we have generated numerical outputs for simulations with various *α* values and noise amplitudes which can be found in Fig. 6, where φ, *ϕ* and the absolute relative error of the *α* estimates are plotted. The curves correspond to 4 different noise amplitudes and every plot is the mean of 50 simulations.

Outside the introgression regime, the mixture coefficient estimates have around 2% relative accuracy. The admixture angle φ, close to $180^{\circ }$, shows that the post-JL reconstruction is of good quality. At odds with this, the *ϕ* estimate points out towards nonadmixture in one-third of the simulations.

The difficulties that the introgression regime poses can also be appreciated. The curves for the admixture angle φ plummet with small *α*. Notice the tendency of φ to worsen as the noise amplitude increases: the post-JL admixture reconstruction is harder for long evolutionary times. Interestingly, all the outcomes correspond to $\varphi >90^{\circ }$, namely, true admixture. The results of the 2-way admixture problem with balanced proportions tend to be less sensitive to the amount of noise.

### Results for 3-way admixture

**Fig. 7.**
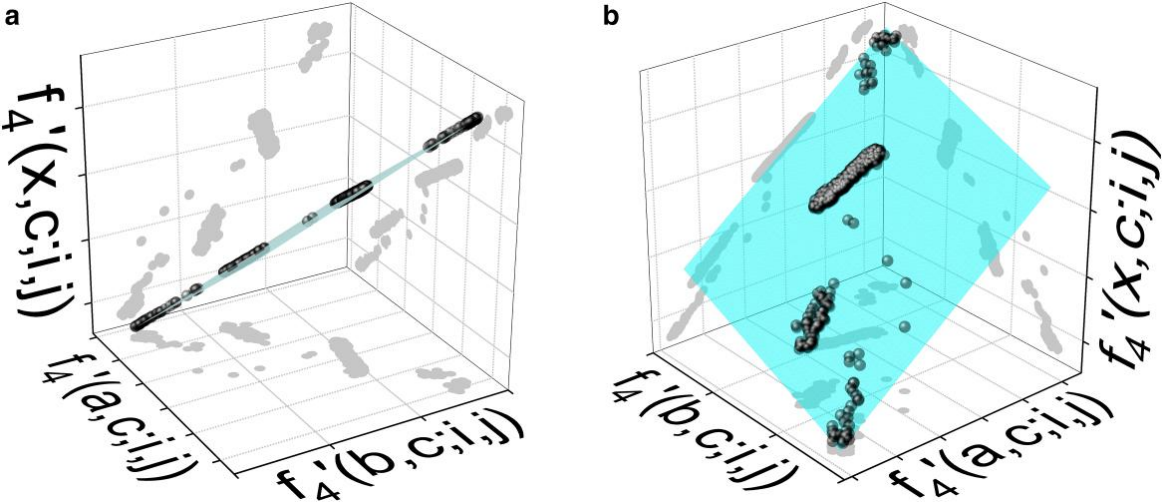
Three-way admixture obtained from α⋅English+β⋅Yoruba+γ⋅Biaka, with nominal α=0.2,β=0.5,γ=0.3. Noise of amplitude ϵ=0.1 has been added to the 4 populations prior to the fit. The numerical fit gives admixture proportions: α=0.18,β=0.54,γ=0.28. Panels (a) and (b) are 2 different perspectives. The gray dots are the orthogonal projections of data. (a) Simulated: English⊕Yoruba⊕Biaka and (b) Simulated: English⊕Yoruba⊕Biaka.

Results for the numerical simulation of 3-way admixtures are displayed in the 4 panels of Fig. 7. The simulated model: 0.2⋅English +0.5⋅Yoruba +0.3⋅Biaka is shown with 2 different perspectives in panels (a) and (b). They exhibit the expected co-planar distribution of points. The flatness index decreases from 0.501 to 0.017, i.e. one order of magnitude.

**Fig. 8.**
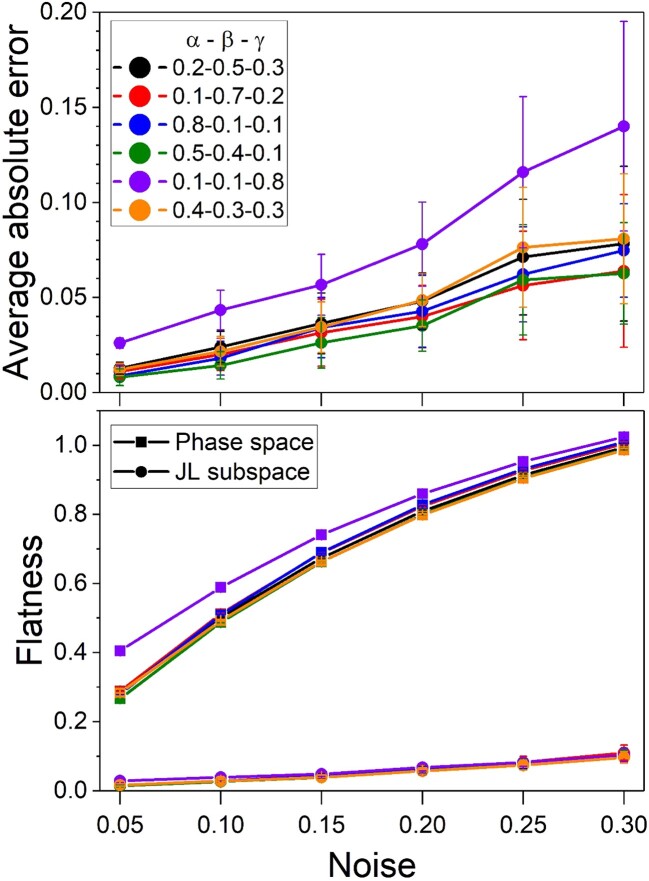
Six 3-way admixture simulation: α⋅English+β⋅Yoruba+γ⋅Biaka, with coefficients in the legend, as a function of noise. The upper panel represents the average of the 3 absolute errors in the mixing proportions. With the same color codification, the bottom panel stands for the flatness index in the allele frequency space (squares) and in the JL subspace (circles). Every point corresponds the average of 50 numerical simulation runs with different noise realization. Error bars are 1 SD.

We have simulated models generated by: α⋅English +β⋅Yoruba +γ⋅Biaka, with six different combinations of mixing proportions, and several noise amplitudes. Fig. 8 gathers the outcomes. Every point stands for an average of 50 simulations. The upper panel shows the average of the 3 absolute errors between the estimate and the nominal α,β,γ. The quality of the estimates worsens with increasing noise, as expected. Moreover, it also depends on the mixing proportion combination. As a rule, more balanced admixtures tend to be better estimated. The bottom panel in Fig. 8 shows the flatness index in the allele frequency space and in the JL subspace, as a function of noise. The flattening of the tetrahedron structure is manifest.

## Discussion

The essence of the geometric perspective for the *f*-formalism approach to the population mixing problem is briefly discussed below.

There is a formal part in which the populations in the dataset are viewed as points in the allele frequency space. One has to decide on the basis of prior information whether to model either a 2- or a 3-way admixture. The proxies of the admixture populations configure a triangle or a tetrahedron in phase space, following the 2- or 3-way case. The auxiliary populations, in turn, define the random subspace where the proxies are projected and, according to the JL lemma, the relative proportions of the triangle or the tetrahedron are approximately preserved. The key point is that, due to the high dimension of the phase space and the random nature of genetic drift, the very JL projections deplete the post-admixture drift. Thus, once projected, the triangle and the tetrahedron become geometric configurations that are close to an admixture at its inception.

In a 2-way admixture, the proxies form a triangle in phase space and the mathematical condition for admixture reads $90^{\circ }<\phi <180^{\circ }$, for the angle at the hybrid vertex. After JL projection, the new triangle should have the vertices almost aligned. The closer the post JL angle φ to $180^{\circ }$, the better the reconstruction of the native admixture configuration. Moreover, numerical experiments have shown that cases with $\phi <90^{\circ }$, and so outside the mathematical admixture domain, can still be successfully mapped to $\varphi ∼180^{\circ }$. This is a new feature of the *f*-formalism that emerges in the geometric picture.

In a 3-way admixture, the proxy samples form an irregular tetrahedron in phase space which after JL projection becomes flatter. The closer to a co-planar configuration, the better the reconstruction will have been. The mapping efficiency is quantified by the heuristic flatness index.

In practice, the task of estimating mixing coefficients and confidence intervals consists in carrying out an appropriate linear fit. We are given a dataset with allele frequencies of a number *n* of populations, for a large number *s* of SNPs. One population is the hybrid and 2, or 3, the contributors. The remaining n−3, or n−4, are used as auxiliary populations. The admixture proportion estimates come from a linear fit through the origin to the set $\left\{f_{2}^{\prime }(a,b;i,j\right)$, $f_{4}^{\prime }(x,b;i,j)\}$ with (n−3)(n−4)/2 points, in a 2-way admixture. In a 3-way admixture, we fit a plane through the origin to the set $\left\{f_{2}^{\prime }(a,c;i,j\right)$, $f_{4}^{\prime }(b,c;i,j)$, $f_{4}^{\prime }(x,c;i,j)\}$ with (n−4)(n−5)/2 points. The fits provide the mixing parameter confidence intervals.

In this regard, an issue left untreated here is the effect that the dispersion of allele frequencies may have on the accuracy of the mixing coefficients estimate. This requires a second dataset where each entry is the error bar estimated for the corresponding allele frequency. The error bars associated with the $\left\{f_{4}^{\prime }\right\}$’s used in the fitting plots could be estimated, e.g. using the error propagation rules. The 2D and 3D plots are the ideal scenario to deal with the situation because the LS solutions can deal with such $f_{4}^{\prime }$ error bars. The technical difficulty is then that error bars affect concomitantly all the axes in the fit.

This finishes the instrumental part. Important remarks should also be made in addition. (a) Two slightly different rationales underpin the geometric and the standard framework derivations. The former consists in a proper projection into a subspace, with unit vectors, and hence $f_{4}^{\prime }$. The latter assumes proportionality of the shared drift, namely $f_{4}$. It can be also interpreted as a JL projection with not normalized vectors. The high dimension effect termed concentration of the mean may explain why this shortcoming has not great influence on 2-way estimates. (b) The introgression regime gets more quickly affected by post-admixture drift. The time to fool the admixture test decreases linearly with the proportion of the introgressor, as compared to more balanced mixtures. (c) Auxiliary populations having large shared drift with donors improve the accuracy of proportion estimates. (d) Auxiliary populations with very small shared drift should not impair the estimates because they accumulate at the graphical representation origin. However, they can give unrealistic determinations in a standalone computation of the $f_{4}$-ratio. These observations may be relevant in unsupervised computations. (e) The numerical simulations we have figured out are restricted to describe only the effect of generic drift in the population admixture problem.

The idea of using a graphical visualization of the population admixture problem may help us better grasp it and foster new applications. In this respect, we have introduced the angles *ϕ* and φ, pre- and post-JL projection, respectively. Whereas the value of *ϕ* is only a primary indicator to witness a population admixture, the value of φ is significant in assessing the reconstruction quality of the native admixture configuration.

On the basis of the Brownian trajectories that populations may have taken in phase space, we may also understand the amplified impact that genetic drift has on introgression in comparison to an even mixture, because the exchange of roles hybrid/donor is more likely. Moreover, the impact of nongenetic drift effects such as sudden jumps in phase space is easily understandable in this picture.

Eventually, we cite an interesting *rule of thumb* proposed in Peter (2022) in connection with the interpretation of PCA plots, commonly used to study population structure. There, the very geometric interpretation of the constraint, $90^{\circ }<\phi <180^{\circ }$, leads to an approximate recipe. In such PCA plots, given 2 admixture contributors, the putative hybrids should be inside the circle whose diameter is determined by the 2 donors.

We end this discussion with the reminder that the population admixture problem has facets other than the post-admixture drift that are less tractable. The geometric view provides fresh perspectives to the essentially statistical foundations of the *f*-formalism that could help us better grasp untackled aspects.

## Supplementary Material

iyae134_Supplementary_Data

## Data Availability

Codes freely available at https://github.com/jaoteo/mixtum. Supplemental material available at GENETICS online.
