# Supplementary material for: The geometry of admixture in population genetics: the blessing of dimensionality
Source: Genetics. 2024 Aug 14;228(2):iyae134. doi: 10.1093/genetics/iyae134 (PMC11639143; doi:10.1093/genetics/iyae134)
Supplement: iyae134_Supplementary_Data [file iyae134_supplementary_data.pdf]

Supplementary document
The geometry of admixture in population genetics: the blessing of dimensionality

José-Angel Oteo and Gonzalo Oteo-García

The details of the algebraic developments that led to the results in the main text are disclosed here as well as additional results that buttress the geometric interpretation of the population admixture problem. Because the presentation is meant to stand alone, certain equations have been repeated for readability.

The three mathematical tools used are described and illustrated: (1) the Johnson-Lindenstrauss (JL) lemma, (2) the quasi-orthogonality in high dimension, and (3) the scalar (or dot) product of two vectors, definition and geometric interpretation.

We commence by reviewing the definitions of the f-formalism, due to its role as the basic notation of the geometric framework. We also describe the phylogenetic meaning of these three statistics.

S.1 The f-formalism: definitions

The three basic f-statistics are usually introduced as correlation measures among the allele frequencies involving two, three or four populations

$$\begin{aligned} f_4(a,b;i,j) &= \frac{1}{s}\sum_{k=1}^{s}(p_a^k - p_b^k)(p_i^k - p_j^k), \\ f_3(a,b;m) &= \frac{1}{s}\sum_{k=1}^{s}(p_a^k - p_m^k)(p_b^k - p_m^k), \\ f_2(a,b) &= \frac{1}{s}\sum_{k=1}^{s}(p_a^k - p_b^k)^2, \end{aligned} \tag{S.1}$$

where p_i^k stands for the allele frequency of population i and SNP k. The number of SNPs is s. Note that f_2 and f_3 are degenerate cases of f_4. Populations a, b and i, j are presently referred to as *left* and *right* populations, respectively, in the literature. Right and auxiliary populations are equivalent.

In the geometric interpretation of the formalism the s allele frequencies $\{p_a^k\}$ of a population a are interpreted as the components of the vector $\vec{p}_a$ in a phase space, the allele frequency space, where every population is represented by a point. This picture leads us to rewrite the three statistics definitions in terms of scalar (or dot) products

$$\begin{aligned} f_4(a,b;i,j) &= \frac{1}{s}(\vec{p}_a - \vec{p}_b)\cdot(\vec{p}_i - \vec{p}_j), \\ f_3(a,b;i) &= f_4(a,i;b;i), \\ f_2(a,b) &= f_4(a,b;a,b). \end{aligned} \tag{S.2}$$

Here, the dot stands for the scalar product of two vectors

$$\vec{u}\cdot\vec{v} = \sum_{k=1}^{s} u^k v^k = |u|\,|v|\,\cos\theta, \tag{S.3}$$

where θ is the angle between vectors $\vec{u}$ and $\vec{v}$, u^k is the k-component of $\vec{u}$ and $|\vec{u}|$ the modulus. Concomitantly, $\vec{u}\cdot\vec{v}$ is the length of the projection of $\vec{v}$ along $\vec{u}$, times the length $|\vec{u}|$. In the following, the components p_a^k of the vector $\vec{p}_a$ are the different allele frequencies of population a in the dataset.

The three f-statistics (S.2) are then scalar products normalized to the number s of SNPs, by definition.

The first, $f_4(a,b;i,j)$, has a phylogenetic meaning. In the phylogenetic tree $(((i,a),b),j)$, it determines the amount of shared drift between population pairs (a,b) and (i,j). f_4 establishes also the algebraic condition for phylogeny of the four populations tree above

$$f_4(i,a;b,j) = 0, \tag{S.4}$$

a result used in the 3-way admixture case. The f_4-statistics is the basis for the so-called f_4-ratio in population admixture which estimates the proportion in a 2-way population admixture.

The second, $f_3(a,b;i)$, stands for the genetic distance between population i and the node (a,b), measured on the phylogenetic tree $((a,b),i)$.

The third, $f_2(a,b)$, is the squared Euclidean distance between a and b in phase space, normalized to s.

A caveat. We use f_2, f_3, and f_4 as a mere notation resource to re-express involved scalar products. We do not resort to their statistical meaning but only to their geometric properties. Of course, the dispersion inherent to the experimental allele frequencies are inherited by the scalar products.

S.2 The geometry of population admixture

Consider the case of two populations thought to be the contributors to a third, hybrid, population. Assume that esti-

mates of the allele frequencies of a number s of SNPs are known. These three populations are the points a, b, x, respectively, in Figure 1, where $s = 3$. They are the proxies of the ancestral populations a', b', x', extant when the admixture takes place. Indeed, the crinkly trajectories separating a, b, x from a', b', x' are the knot of the population admixture problem. It is important to note that the diagram hosts a number of features that cannot be consistently represented in 3D. For instance, the points structure $\{a', x', b', b, x, a\}$ is by no means planar because vectors from a' to a, from x' to x, and from b' to b, point towards arbitrary directions. Moreover, the genetic drift that drives population b' to b is such that the segment $\overline{bb'}$ is approximately orthogonal to $\overline{a'b'}$, a fact that is indicated by the squared (green) angle. There exist further quasi-orthogonality (denoted $\perp$) constraints: $\overline{aa'} \perp \overline{bb'} \perp \overline{xx'} \perp \overline{a'b'}$. This quasi-orthogonality-based description, which is justified below, is a consequence of the dynamics induced by genetic drift in a high dimension space.

The population vectors $\vec{p}$ in phase space, whose components are the allele frequencies, lie only in the positive orthant of the phase-space and $|\vec{p}| \leq s$, because allele frequencies are positive numbers smaller that unit. The hybridization process is assumed to take place suddenly in evolutionary terms so that the vector $\vec{p}_{x'}$ of an admixed population x' is then a linear combination, in the vector algebra sense, of the allele frequency vectors of the donors. For a 2-way admixture process we get $\vec{p}_{x'} = \alpha \vec{p}_{a'} + (1-\alpha)\vec{p}_{b'}$, with $\alpha \in [0, 1]$ the contributing fraction of population a' [2, 8]. However, no experimental information about primed populations is usually available and we have to pose the linear combination with the proxy populations

$$\vec{p}_x = \alpha \vec{p}_a + (1-\alpha)\vec{p}_b, \quad \alpha \in [0, 1], \tag{S.5}$$

which is only exact at the inception of the admixed population.

Mathematically, the linear combination (S.5) stands for an over-determined linear algebraic system of dimension s with only one unknown, α, that can estimated in the sense of Least Squares (LS). The LS outcome can be readily written down in terms of scalar products of allele frequency vectors to give

$$\begin{aligned} \alpha &= \frac{(\vec{p}_x - \vec{p}_b) \cdot (\vec{p}_a - \vec{p}_b)}{(\vec{p}_a - \vec{p}_b)^2}, \\ &= \frac{f_3(a, x; b)}{f_2(a, b)}. \end{aligned} \tag{S.6}$$

This is the slope of the best fitting line through the origin to the set $\{p_x^i - p_b^i, p_a^i - p_b^i\}$, $i = 1, \ldots, s$. One could instead use a standard routine to fit a straight line through the origin to the set of s points to determine the slope α and its confidence interval.

It is straightforward to obtain, from (S.5), the constraint

$$(\vec{p}_x - \vec{p}_a) \cdot (\vec{p}_x - \vec{p}_b) = -\alpha(1-\alpha)(\vec{p}_a - \vec{p}_b)^2, \quad \alpha \in [0, 1], \tag{S.7}$$

which conveys that the left-hand-side must be negative and therefore the angle ϕ between vectors $(\vec{p}_x - \vec{p}_a)$ and $(\vec{p}_x - \vec{p}_b)$ obtuse, according to (S.3). This is the mathematical condition for admixture and in terms of scalar products reads

$$\begin{aligned} \cos\phi &= \frac{(\vec{p}_x - \vec{p}_a) \cdot (\vec{p}_x - \vec{p}_b)}{[(\vec{p}_x - \vec{p}_a)^2 \ (\vec{p}_x - \vec{p}_b)^2]^{1/2}}, \\ &= \frac{f_3(a, b; x)}{[f_2(a, x) f_2(b, x)]^{1/2}} < 0. \end{aligned} \tag{S.8}$$

Since the denominators are positive we get $f_3(a, b; x) < 0$, the current admixture test.

The geometric interpretation of the α estimate (S.6) goes through the admixture triangle $\triangle axb$ in Figure 2b. The α value corresponds to the ratio p/q whose numerator is the length of the orthogonal projection p of the side $\overline{xb}$ onto the side $\overline{ab}$, and the denominator is the side length $|\overline{ab}| = q$. By now, this straightforward approach does not appear to benefit from the problem's high dimensionality considerations.

Let us consider k auxiliary populations i, j, as those represented in the Figure 1. The difference vectors $\vec{p}_i - \vec{p}_j$ define arbitrary directions in phase space. Projecting the system (S.5) along them leads to the overdetermined system

$$\frac{(\vec{p}_x - \vec{p}_b) \cdot (\vec{p}_i - \vec{p}_j)}{\sqrt{(\vec{p}_i - \vec{p}_j)^2}} = \alpha \, \frac{(\vec{p}_a - \vec{p}_b) \cdot (\vec{p}_i - \vec{p}_j)}{\sqrt{(\vec{p}_i - \vec{p}_j)^2}}, \; i < j = 1, \ldots, k, \tag{S.9}$$

with $d = k(k-1)/2$ equations and one unknown, α. The denominator ensures that the projection is properly done with unit vectors: $(\vec{p}_i - \vec{p}_j)/[(\vec{p}_i - \vec{p}_j)^2]^{1/2}$. This system can be rewritten as

$$f_4'(x, b; i, j) = \alpha \, f_4'(a, b; i, j), \quad i < j = 1, \ldots, k, \tag{S.10}$$

in terms of *renormalized* f_4-statistics

$$f_4'(a, b; i, j) = \frac{(\vec{p}_a - \vec{p}_b) \cdot (\vec{p}_i - \vec{p}_j)}{[(\vec{p}_i - \vec{p}_j)^2]^{1/2}} = \frac{\sqrt{s}\, f_4(a, b; i, j)}{\sqrt{f_2(i, j)}} \tag{S.11}$$

We can see (S.9) as the system (S.5) projected into a random subspace of dimension d and, continuing the game, the LS solution reads

$$\begin{aligned} \alpha &= \frac{\sum_{i<j}^{k} [(\vec{p}_x - \vec{p}_b) \cdot (\vec{p}_i - \vec{p}_j)][(\vec{p}_a - \vec{p}_b) \cdot (\vec{p}_i - \vec{p}_j)]/(\vec{p}_i - \vec{p}_j)^2}{\sum_{i<j}^{k} [(\vec{p}_a - \vec{p}_b) \cdot (\vec{p}_i - \vec{p}_j)]^2/(\vec{p}_i - \vec{p}_j)^2}, \\ &= \frac{\sum_{i<j}^{k} f_4'(x, b; i, j) f_4'(a, b; i, j)}{\sum_{i<j}^{k} f_4'^2(a, b; i, j)}. \end{aligned} \tag{S.12}$$

As with (S.6), equation (S.12) is the slope of a fit of a line through the origin to the d points of set $\{f_4'(a, b; i, j), f_4'(x, b; i, j)\}$. In practice, a numerical routine could be alternatively used to compute the slope and the confidence interval.

Had we only one pair of auxiliary populations, then

$$\alpha = \frac{f_4(x, b; 1, 2)}{f_4(a, b; 1, 2)}, \tag{S.13}$$

which is the so-called f_4-ratio [9].

Indeed, the projection of the linear system (S.5) along the arbitrary directions $\vec{p}_i - \vec{p}_j$ appears to be a whimsical procedure, raising the question: how much does the α estimate depend on the auxiliary populations choice? Or, what is the gain of using (S.12) instead of (S.6)? To address these and other issues some needed mathematical considerations [11] are explained next. The random projection (S.9) will prove to be an effective strategy and a basic element in the f-statistics approach to the population admixture problem.

S.3 Interlude: the blessing of dimensionality

The Johnson-Lindenstrauss lemma provides the first mathematical element we require. It states that a set of m points in a large dimensional space $\mathbb{R}^n$ may be projected into a random subspace $\mathbb{R}^d$, with $d \ll n$, such that the pairwise distances of the point set are approximately preserved. The interesting feature is that for a given pairwise distance accuracy the reduced dimension d is independent of the original dimension n. Only the number of points m has an impact.

Lemma 1: Johnson-Lindenstrauss. Given $\epsilon \in (0, 1/2)$, then for any set of points $S = \{x_1, \ldots, x_m\}$ in $\mathbb{R}^n$, there exists the mapping $M : \mathbb{R}^n \to \mathbb{R}^d$ with $d = \mathcal{O}(\log(m)/\epsilon^2)$ such that

$$\forall x_1, x_2 \in S : ||x_1 - x_2||(1-\epsilon) \leq ||M\, x_1 - M\, x_2|| \leq ||x_1 - x_2||(1+\epsilon).$$

In algorithmic practice the mapping M is a $d \times n$ random matrix. We will associate the rows of this matrix to the difference vectors of auxiliary populations that carry out the projections in (S.9).

The second element refers to the quasi-orthogonality of random vectors in high dimension.

Lemma 2: quasi-orthogonality in high dimension spaces. Let $\vec{u}$ be a unit vector in R^n, and $\vec{z} = (z_1, \ldots, z_n)/\sqrt{n} \in R^n$, with each coordinate z_i at random from $[-1, 1]$. If θ is the angle between $\vec{u}$ and $\vec{z}$, then we have the following bound on the probability

$$Pr(|\cos\theta| > \epsilon) < \exp(-n\,\epsilon^2).$$

The amount of quasi-orthogonal random vectors grows exponentially with the space dimension. Figure S.1 captures this phenomenon showing the histogram of the values of the $\cos\theta$ between the vector $(1, \ldots, 1)$ and $\vec{z}$ for four different values of the space dimension. The histogram shrinks as far as the dimension increases indicating quasi-orthogonality ($\cos\theta \simeq 0$).

In data mining, the JL lemma is used to reduce the numerical burden associated with large datasets. In our case the true utility stems from the involved projections and from the quasi-orthogonality property, which bring the proxies closer to the ancestral population admixture configuration in the allele frequency space by removing the post-admixture drift contribution. Given the populations a, x, b in Figure 1, we introduce the vector decomposition

$$\vec{p}_a = \vec{p}_{a'} + \vec{g}_a, \quad \vec{p}_x = \vec{p}_{x'} + \vec{g}_x, \quad \vec{p}_b = \vec{p}_{b'} + \vec{g}_b, \qquad \text{(S.14)}$$

where $\vec{g}_a, \vec{g}_x, \vec{g}_b$, stand for the post-admixture drift contributions to the ancestral populations with each coordinate at random, with the proviso that allele frequencies are in range. Next, we analyze the two scalar products in the numerators of (S.9) as regards quasi-orthogonality. For one of them

$$\begin{aligned}(\vec{p}_a - \vec{p}_b)\cdot(\vec{p}_i - \vec{p}_j) &= (\vec{p}_{a'} - \vec{p}_{b'} + \vec{g}_a - \vec{g}_b)\cdot(\vec{p}_i - \vec{p}_j)\\ &\simeq (\vec{p}_{a'} - \vec{p}_{b'})\cdot(\vec{p}_i - \vec{p}_j), \qquad \text{(S.15)}\end{aligned}$$

because lemma 2. And similarly for the other numerator. As a result, everything happens (approximately) in (S.9) as if we were working with ancestral populations rather than proxies. This is a feature already pointed out in the f-formalism using statistical foundations [9].

Using only one pair of auxiliary populations, namely i and j fixed, implies a drastic dimensional reduction of the equation system (S.5) from dimension $s \gg 1$ to a subspace of dimension $d = 1$, defined by the vector $\vec{p}_i - \vec{p}_j$; and therefore (S.9) collapses to one scalar equation. In the light of JL lemma, $d = 1$ must convey a significant distortion. By letting i, j be $k > 2$ running auxiliary populations, we define a higher dimension subspace of dimension $d = k(k-1)/2$. The auxiliary pairs then define arbitrary directions to form the vector basis of a subspace, say the JL subspace. We expect less distortion after projection with k large enough (i.e., $d > 1$) than with $k = 2$ (i.e., $d = 1$).

With $m = 3$ or 4 admixing populations, the JL lemma establishes that the subspace dimension d goes with $1/\epsilon^2$. Consequently, the number of auxiliary populations k goes as $1/\epsilon$. In particular, for distortion $\epsilon = 0.01$, we get $k \simeq 150$ auxiliary populations in 2-way and 3-way admixture. At this point, it is convenient remembering that the bounds in the lemma are not necessarily optimal because m is arbitrary. Small m values could lead to lower bounds.

It is expected the approximate preservation of pairwise distances between vertices, combined with the approximate suppression of post-admixture drift contributions, to transform the phase space triangle $\triangle abc$ into a more obtusangle triangle $\tilde{\triangle} abc$ in the JL subspace, namely, a configuration close to an admixture at its inception. It turns out that the very rule (S.6), when applied in the JL subspace, becomes (S.12), as explained next.

S.4 One triangle to rule them all

Given that the set $\{(\vec{p}_i - \vec{p}_j)/|\vec{p}_i - \vec{p}_j|\}$ is a quasi-orthonormal vector basis, the sums in (S.12) represent scalar products in the JL subspace, in good approximation. This is because $f'_4(a, b; i, j)$ is (up to a constant) the JL subspace component of $(\vec{p}_a - \vec{p}_b)$ along the direction given by $(\vec{p}_i - \vec{p}_j)$ and, hard to believe, the sums in (S.12) are (approximate) scalar products in the JL subspace with exactly the same interpretation as (S.6).

The advantage of (S.12) is that the vertices of the JL projected triangle $\tilde{\triangle} axb$ are expected to be almost a linear alignment, the characteristic of an admixture at its inception.

Moreover, we can replicate this reasoning to estimate the angle φ at the vertex x of the JL projected triangle $\tilde{\triangle} axb$:

$$\cos\varphi = \frac{\sum_{i<j}^{k} f'_4(x, a; i, j) f'_4(x, b; i, j)}{[\sum_{i<j}^{k} f'^2_4(x, a; i, j) \sum_{i<j}^{k} f'^2_4(x, b; i, j)]^{1/2}}, \qquad \text{(S.16)}$$

to be compared with $\cos\phi$ in (S.8). Here, use has been made of the identity (S.3). As far as $\cos\varphi \ll \cos\phi$, or more precisely, as far as $\varphi \to 180°$, it is expected (S.12) to improve the α estimate with respect to the determination (S.6), obtained in the full allele frequency space. The closer φ to 180°, the better the JL reconstruction of the admixture model at its inception.

S.5 The f_4-ratio illustrated

As already pointed out in Section *Remarks about the estimation of* α, the geometric interpretation warns about the standalone use of the f_4-ratio. Here is why.

The evaluation of the f_4-ratio (S.13) on its own goes through two projections in the triangle $\triangle axb$ of Figure 1. The numerator and denominator are the projection of the sides xb and ab, respectively, onto a one dimensional subspace defined by the difference vector of two auxiliary populations, $\vec{p}_i - \vec{p}_j$. The geometry of such an evaluation is sketched in Figure S.2 where, besides the triangle, four different one dimensional projections have been depicted. The diagram is planar only for the sake of clarity. The dashed segments $\overline{ij}$, $\overline{i'j'}$ and $\overline{i''j''}$ stand for directions defined by the auxiliary pairs: $\vec{p}_i - \vec{p}_j$, $\vec{p}_{i'} - \vec{p}_{j'}$ and $\vec{p}_{i''} - \vec{p}_{j''}$. Gray lines are eye guides for the following orthogonal projections:

1. Projection on $\overline{ab}$. The sides $\overline{xb}$ and $\overline{ab}$ yield projection segments of lengths p (blue) and q (red), respectively; and $\alpha = p/q \in [0, 1]$, always in range under mathematical admixture conditions. This describes case (S.6), and is not properly an f_4-ratio.

2. Projection on $\overline{ij}$. The sides $\overline{xb}$ and $\overline{ab}$ yield projection segments of lengths s (blue) and r (red), respectively. The corresponding f_4-ratio yields the estimate $\alpha = r/s > 1$, out of range.

3. Projection on $\overline{i'j'}$. The sides $\overline{xb}$ and $\overline{ab}$ yield projection segments of lengths t (red) and u (blue), respectively; and $\alpha = t/u \in [0, 1]$, in range.

4. Projection on $\overline{i''j''}$. The sides $\overline{xb}$ and $\overline{ab}$ yield projection segments of lengths y (red) and z (blue), respectively; and $\alpha = -y/z < 0$, out of range.

Projection#1 does not belong to the f_4-ratio realm. It is given for the sake of completeness. It always leads to $\alpha \in [0, 1]$, provided the admixture condition holds, namely $f_3(a, b; x) < 0$. In cases with small post-admixture drift, which conveys an angle close to 180° at vertex x, equation (S.6) should provide an accurate determination. This scenario takes place in recent population mixtures.

Projections #2, #3 and #4 are characteristic of the f_4-ratio procedure. Projection#3 gives a mathematically admissible estimate, whereas projection#2 and projection#4 fail to be in range. In view of the JL lemma, it is understandable that the drastic dimensional reduction to dimension one has caused a great distortion in the projection. This is illustrated in Figure S.3 where the results of calculating the f_4-ratio for each auxiliary pair are shown in combination with the shared drift in absolute value. Figure S.3a illustrates, with the simulated model: 0.1·English+0.9·Yoruba, that little shared drift leads commonly to unreliable f_4-ratio estimates, a fact that the linear regression avoids. Concomitantly, for the model: Italian_North = α·Basque +$(1-\alpha)$·Adygei, Figure S.3b shows that unrealistic population admixtures lead to f_4-ratio estimates of broad spectrum. The zoomed area in the inset has a similar scale to Figure S.3a, for the sake of comparison. Both plots point out a concentration of the f_4-ratio values wherever the shared drift is large, albeit the dispersion is much greater for the unrealistic admixture.

S.6 Two-way admixture: the f-statistics standard way

The standard f-formalism does not have currently an explicit formula for the α estimate of the 2-way admixture. It is possible to track the analytic computations up to a point [3]. Let us rewrite the linear combination (S.5) as

$$\vec{p}_x = \alpha\vec{p}_a + \beta\vec{p}_b, \quad \alpha + \beta = 1, \ \ \alpha, \beta \in [0, 1]. \tag{S.17}$$

Then, due to the linear character of the scalar product we get

$$\alpha f_4(x, a; i, j) + \beta f_4(x, b; i, j) = 0, \quad \alpha + \beta = 1, \tag{S.18}$$

for any pair of auxiliary populations i, j. Considering $d = k(k-1)/2$ such pairs we get an overdetermined linear system with two unknowns and one constraint that can be formally written down in matrix form

$$\vec{w}.C = 0, \quad \alpha + \beta = 1, \quad \vec{w} = (\alpha, \beta), \tag{S.19}$$

where $C \in \mathbb{R}^{2\times d}$. A solution of LS type could be worked out at this point. Instead, the matrix C is decomposed via Singular Value Decomposition as: $C = A.B$. This is the seed for an iterative process that minimizes the log-likelihood for (A, B) and takes into account the error introduced by the SNP sampling via a block jackknife resampling procedure. It is clear that no analytic expression can be given beyond this point. The admixture proportions are then found as the LS solution of

$$\vec{w}.A = 0, \quad \alpha + \beta = 1, \quad \vec{w} = (\alpha, \beta), \tag{S.20}$$

where $A \in \mathbb{R}^{2\times d}$ is the result of an elaborated procedure. The special purpose *AdmixTools* package, and particularly the *qpAdm* function, has been designed for this task [3, 9, 5].

The LS solutions of (S.19) and (S.20) can be compared on an equal footing if the impact of the SNP sampling is not taken into account. Under this assumption, (S.18) lends us a comparison starting point with the results in Section *Practical results: two-way admixture*. It can be rewritten as

$$f_4(x, b; i, j) = \frac{\alpha}{\alpha - 1} f_4(x, a; i, j), \quad i < j = 1, \ldots, k, \tag{S.21}$$

once the constraint has been explicitly introduced into the equations. Linear plots with this relationship are used in [4, Supplementary information]. A little algebra yields the simpler system

$$f_4(x, b; i, j) = \alpha f_4(a, b; i, j), \quad i < j = 1, \ldots, k, \tag{S.22}$$

to be compared with (S.10) or (5). Now, according to the phylogenetic interpretation of f_4 as shared drift, this relationship tells us that given the tree $(((i, a), b), j)$ and the hybrid x with donors a and b, the shared drift between (x, b) and (i, j) must

be proportional to the one between (a,b) and (i,j). And α is the proportion of admixture contributed by population a. The problem is then algebraically similar to that in (S.10) or (5). In a 2D representation, the set of values $\{f_4(a,b;i,j), f_4(x,b;i,j)\}$, with $i<j=1,\ldots,k$, should appear close to a straight line with slope α, as far as the admixture model is correct. The explicit LS formula to estimate the slope reads

$$\alpha = \frac{\sum_{i<j}^{k} f_4(x,b;i,j) f_4(a,b;i,j)}{\sum_{i<j}^{k} [f_4(a,b;i,j)]^2}, \tag{S.23}$$

which is the unprimed equation (S.12), or (6). The f_4-ratio definition (S.13) is recovered for i,j fix.

Let us analyze the rationales that have led to slightly different equations (S.12) and (S.23). Both, the geometry-based scheme and the standard f-formalism, assume the same admixture proportion for every single SNP, i.e. equation (S.5). It is the way the JL projection is done what makes the difference of both schemes. Technically, a geometric projection requires unit length vectors. This is why the primed f_4' emerges. The proportionality of the shared drift in (S.10) between the pairs (a,b) and (i,j) with respect to the pairs (x,b) and (i,j) gets weighted by the renormalized Euclidean distance in the allele frequency space between populations i and j: the further away, the less relative importance has that JL component. The standard f-derivation goes with (S.22) and the proportionality of the shared drift is unweighted by $f_2(i,j)$. We could interpret it as a JL-like projection which does not use unit base vectors. Therefore, unless all vectors $\vec{p}_i - \vec{p}_j$ have similar modulus, the projection will suffer from a certain degree of distortion. A formal justification that alleviates this issue relies on a high-dimensional effect known as *concentration of the norm* [10]. It establishes that the norm of random vectors generated from the same distribution tends to a fixed value. In other words, the vector tips cover approximately a hyper-sphere. Whether or not the vectors $\vec{p}_i - \vec{p}_j$ fit strictly the mathematical requirements, the outcomes of (S.10) and (S.22) make little difference as regards the α determination according to the numerical simulations.

S.7 The quasi-orthogonality of the dataset

The quasi-orthogonality of the set of auxiliary populations is a fundamental piece of the framework. Thus, the question of how much orthogonal the auxiliary population set is arises. For the forty population dataset in the main text the answer is in Figure S.4 (bottom panel) which presents a histogram of the absolute value of the cosine of the angle between two population pairs. The distribution is peaked at 90°.

To buttress that this feature is not a coincidence we have generated a similar histogram associated to the so-called auxiliary population dataset **O9** [6]: Ust_Ishim, Kostenki14, MA1, Han, Papuan, Onge, Chukchi, Karitiana and Mbuti. This gives 36 pairs of auxiliary populations and the effective number of SNPs is in this case $s = 380.574$. The populations in this set have been carefully chosen in [6] according to their location in a phylogenetic tree, in contrast with the choice of populations in the forty populations dataset which was rather whimsical. The quasi-orthogonality of the **O9** set is disclosed in the histogram of Figure S.4 (top panel).

The distributions in these two panels give a glimpse about the practical meaning of quasi-orthogonality.

S.8 Standard f_4 versus renormalized f_4' representations

The observed equivalence of the 2D representations, f_4 and f_4', with respect to the α estimation points out that the implicit denominators in the equation system (S.10) cannot have a great relative diversity in magnitude. This is witnessed in the histogram of the Euclidean distance of the population pairs in the dataset given in Figure S.5. The Euclidean distance of the bulk of pairs is in the range 144 ± 50, and the tails of the distribution are small. The variability of denominators is smaller than one order of magnitude.

S.9 The choice of auxiliary populations

Consider the unrooted phylogenetic graph in Figure 3. Besides the admixture triplet there are five auxiliary populations termed d_i (by *dextra*) and s_i (by *sinistra*), according to their visual location with respect to the admixture triplet in the diagram, and w is a population located between a and x. The idea is to discuss the effect that different choices of auxiliary population pairs have in connection with their relative phylogenetic location.

Choosing sinistra-dextra auxiliary pairs provides points $\{f_4(x,b;s_i,d_j), f_4(a,b;s_i,d_j)\}$ $(i,j=1,2)$, that are not close to the origin in the 2D fitting plot because they correspond to non-vanishing shared drift: $p = f_4(a,b;s_i,d_j)$ in Figure 3. By contrast, combinations dextra-dextra and sinistra-sinistra lead to vanishing $f_4(a,b;s_1,s_2)$, $f_4(a,b;d_1,d_2)$, $f_4(x,b;s_1,s_2)$, and $f_4(x,b;d_1,d_2)$, as a consequence of lack of shared drift or, in geometric terms, quasi-orthogonality of the auxiliary pairs with respect to the admixture triplet. In practice, these points appear near the origin in the fitting plot. All these are the harmless cases in the linear fitting procedure. However, pairs sinistra-w generate an artifact in the 2D plot because $f_4(a,b;s_i,w) \neq 0$ and $f_4(x,b;s_i,w) = 0$, which corresponds to a point on the horizontal axis of the plot. This situation takes place because the particular form of linear combination (S.5). In case a number of these auxiliary population pairs are to be used such fitting artifacts could be avoided using instead the more symmetric parametrization

$$\vec{p}_x = \frac{1}{2}\left[(1+\beta)\vec{p}_a + (1-\beta)\vec{p}_b\right], \quad \beta \in [-1,1], \tag{S.24}$$

with $\alpha = (1+\beta)/2$. It leads to replace (S.10) with the equivalent

$$\frac{1}{2}\left[f_4'(x,a;i,j) + f_4'(x,b;i,j) + f_4'(a,b;i,j)\right] = \alpha\, f_4'(a,b;i,j), \tag{S.25}$$

with $i < j = 1, \ldots, k$. The idea is to plot the left-hand-side versus $f_4'(a, b; i, j)$ and estimate the slope. Note that the first two terms in the left-hand-side have opposite signs, a feature that may be used to detect this kind of auxiliary population pair in unsupervised computations.

The desideratum for a good auxiliary population pair set is a paradoxical interplay result of two features. First, a histogram as in Figure S.4 should have a short tail. The shorter the tail, the better the quality of the base of the JL subspace. Second, the corresponding histogram with the dataset enlarged to include the involved populations in the admixture model should have a larger tail compared to the former. The more points in the new piece of the histogram tail, the better the slope(s) estimates because this excess of tail is originated by shared drift.

S.10 Difficulties with introgression

When the contribution at admixture inception of (say) donor b' is tiny, i.e. $\alpha \simeq 1$, complex situations are likely to arise in which genetic drift leads to misleading population configurations in phase space. This introgression situation is depicted in both panels of Figure S.6 where the inception of the population admixture corresponds to the segment $\overline{a'x'b'}$ and it is meant that the contribution of b' is tiny. The genetic drift is represented by the Brownian-like trajectories that drive a', x', b' to the triangle $\triangle axb$ in phase space.

In Figure S.6a the initial hybrid x' and the initial donor a' are driven by genetic drift to the wrong vertex in the triangle $\triangle axb$, in the sense that the main contributor a appears as a hybrid and the introgressor x as a donor, according to the admixture geometric condition.

In Figure S.6b the Brownian-like trajectories are such that all the angles in triangle $\triangle axb$ are acute, indicating phylogenetic treeness rather than population admixture.

Both scenarios are likely to take place in long enough evolutionary histories. However, the closeness between a' and x' shorten the time lapse to occur. This qualitative observation can be bounded. The shortest trajectories that lead to the scenarios of Figure S.6 are when a' and x' meet at midway of the segment $\overline{a'b'}$ in phase space. The squared distance covered by both a' and x' is $(\vec{p}_x - \vec{p}_a)^2/4 = (1-\alpha)(\vec{p}_a - \vec{p}_b)^2/4$. We can compare two population admixtures with the same three populations and different proportions, α_0 and α, on the basis that the average squared distance covered in a Brownian motion is proportional to the time elapsed. Let t_0 and t be those time intervals. A lower bound for the relative time in which the admixture test is not fooled reads then

$$\frac{t}{t_0} = \frac{1-\alpha}{1-\alpha_0} \tag{S.26}$$

If we compare introgression ($1 - \alpha \ll 1$) with respect to a balanced admixture (say, $\alpha_0 \simeq 0.5$), it turns out that $t \ll t_0$: the time interval to fool the admixture test is much shorter in introgression than in a balanced admixture.

To what extent, in an introgression scenario, the JL projection will yield a proper admixture triangle $\tilde{\triangle} axb$ in the subspace is an open question. To go beyond (S.26), which provide only relative outcomes, specific experimental data knowledge is required. We need several lineages that are dated at different times and then check the generalized Brownian law for the linear distance d traveled in the allele frequency space in several time intervals. This is to say, $d = \rho t^\sigma$. For normal Brownian trajectory, $\sigma = 1/2$. If the experimental data appear in a $\log - \log$ plot close to a straight line then the slope is the σ estimate and the vertical intercept is $\log \rho$. This could be the calibration method for the relationship between the Brownian wandering of populations in the allele frequency space and the time invested. Equipped with this knowledge, the distances in the allele frequency space are mapped onto time intervals and it is then possible to address absolute measurements about, e.g., lower bounds to fooling the admixture test. In view of the existing long term correlations across the DNA sequences, the so-called linkage disequilibrium, it is possible to get effective estimates $\sigma > 1/2$, as discussed in [7]. This is usually termed fractional Brownian motion, however it may go by other names as well.

S.11 Two-way admixture simulations

In addition to the simulations presented in the main text, two types of numerical simulations are gathered here. Firstly, we consider the model 0.1·English + 0.9·Yoruba, with large noise amplitude $\epsilon = 0.5$; namely a long time-evolution, in order to enhance the misalignment of points in 2D plots. Secondly, we illustrate the behavior of two unlikely real data admixture models.

Figure S.7 shows the outcomes of the linear relationship suggested by (6) and the unprimed version (S.10). The solid line has slope 0.1, the nominal admixture proportion, whereas the fits give estimates $\alpha = 0.105$ and 0.106 for the f_4' and f_4 representations. The admixture proportions are given with the number of significant digits as given by the fitting routine. The angle at the hybrid vertex in full phase space is $\phi = 67.9°$ (hence $f_3 > 0$), which breaks the classical admixture condition. Even though, in the JL subspace the projected angle reads $\varphi = 175.8°$, which witnesses a good reconstruction of the geometric configuration at the time of admixture inception because it is close to 180°. The inset allows to appreciate, for points with small shared drift, that the slopes defined by every single point yield a large variety of f_4-ratio evaluations, most of then out of range.

S.12 Three-way admixture simulations

Consider the 3-way model linear combination proposed in (10)

$$\vec{p}_x = \alpha\vec{p}_a + \beta\vec{p}_b + (1-\alpha-\beta)\vec{p}_c, \quad \alpha, \beta \in [0,1]. \tag{S.27}$$

This linear combination of population vectors in the allele frequency space may be viewed as an over-determined system of dimension s with two unknowns, α, β. Similarly to (S.6), the

LS estimate can be expressed in terms of f-statistics

$$\alpha = \frac{f_2(b,c)f_3(x,a;c) - f_3(a,b;c)f_3(x,b;c)}{f_2(a,c)f_2(b,c) - f_3^2(a,b;c)}$$
$$\beta = \frac{f_2(a,c)f_3(x,b;c) - f_3(a,b;c)f_3(x,a;c)}{f_2(a,c)f_2(b,c) - f_3^2(a,b;c)} \quad \text{(S.28)}$$

Alternatively, one could take a routine to fit in 3D a plane through the origin to the set of s points $\{p_a^i - p_c^i, p_a^i - p_c^i, p_x^i - p_c^i\}$. The outcome are the (S.28) estimates for α and β, and their confidence intervals.

These formulas will work as far as post-admixture drift has not played its role. Thus, the safe alternative is applying the golden rule. After JL projection with $d = k(k-1)/2$ pairs of auxiliary populations the system (S.27) reads

$$f_4'(x,c;i,j) = \alpha f_4'(a,c;i,j) + \beta f_4'(b,c;i,j), \; i < j = 1,\ldots,k, \quad \text{(S.29)}$$

Exactly as described above for the full phase space, a way to look at the LS solution of the system consists in finding the best fit in 3D to a plane through the origin with the d points in the set $\{f_4'(a,c;i,j),\, f_4'(b,c;i,j),\, f_4'(x,c;i,j)\}$. Explicit formulas for α and β are

$$\alpha = \frac{1}{\Delta}\left[\sum_{i<j}[f_4'(b,c;i,j)]^2 \sum_{i<j} f_4'(a,c;i,j)f_4'(x,c;i,j) - \sum_{i<j} f_4'(a,c;i,j)f_4'(b,c;i,j)\sum_{i<j} f_4'(b,c;i,j)f_4'(x,c;i,j)\right],$$
$$\beta = \frac{1}{\Delta}\left[\sum_{i<j}[f_4'(a,c;i,j)]^2 \sum_{i<j} f_4'(b,c;i,j)f_4'(x,c;i,j) - \sum_{i<j} f_4'(a,c;i,j)f_4'(b,c;i,j)\sum_{i<j} f_4'(a,c;i,j)f_4'(x,c;i,j)\right],$$
$$\Delta = \sum_{i<j}[f_4'(a,c;i,j)]^2 \sum_{i<j}[f_4'(b,c;i,j)]^2 - \left[\sum_{i<j} f_4'(a,c;i,j)f_4'(b,c;i,j)\right]^2. \quad \text{(S.30)}$$

In practice, again, one can use a fitting routine to get the two parameter estimates and their confidence interval.

A close look at (S.30) shows that its algebraic structure is equivalent to (S.28). For the same reason exposed in Section S.4 $f_4'(a,b;i,j)$ is the vector component of $(\vec{p}_a - \vec{p}_b)$ in the JL subspace along the subspace direction defined by $(\vec{p}_i - \vec{p}_j)$. Thus, (S.28) and (S.30) stand for the same combination of scalar products, the former in the full phase space and the latter in the JL subspace. Always with the proviso that the JL projection relies on the quasi-orthogonality of the auxiliary population set and therefore introduces a degree of approximation.

As pointed out in the main text we have introduced the dimensionless flatness index $h/\sqrt{A} = 3V/A^{3/2}$. The tetrahedron volume and the area have been calculated with the Cayley-Menger determinant and the Heron formula, respectively [10]; which use just the edge lengths. Explicit formulas are given in Section S.16

S.13 Application to real data

We illustrate the formalism with real-world examples of well-known admixed modern populations as well as negative examples of made-up admixtures. The cases with real data were chosen in connection with the ADMIXTURE plot in Figure 2a of [1]. The two- and three-way admixture outcomes are in Tables S.1 and S.2, respectively. In Table S.1, the two last columns give the pre-JL and post-JL angles as their ratio with respect to the value 180°, which corresponds to the geometry of admixture at inception.

We show that the Afro-Caribbean (ACB) population of the 1000 Genomes Project [1] is validated as the product of a two-way admixture between a European source (GBR) and an African source (YRI). We obtain similarly good results when modelling Mexicans (MXL), another well-known admixed and heterogeneous population, as a mixture of Spaniards (IBS) and Peruvians (PEL). For both these examples, Table S.1, the conventional f_3-test (full phase space) returns negative values and, equivalently, the pre-JL angles are larger than 90°. The accuracy of the reconstructions pointed out by the post-JL angles (96.7% and 96.1%, respectively) indicate these models are fine estimations of the real admixture scenarios.

Figure S.8 shows the plot for the model ACB hybrid of GBR and YRI. The black circles and the red squares correspond to computations with the forty and the O9 auxiliary population sets, respectively. The number of SNPs in **O9** is about two thirds of the large set. Notice that the range of shared drift, given by the horizontal axis, is very different in both analysis. The two linear fits provide the same slope value, 0.11.

The model for the hybrid MXL presents a similar agreement (not shown).

The inset in Figure S.8 is a histogram of f_4-ratio values, with mean, 0.11, and standard deviation, 0.05, obtained with the values that fall within the range $[0, 1]$

For the four made up admixture scenarios in Table S.1 the f_3-tests return positive values, and the pre-JL angles are smaller than 90°, indicating no admixture, at first. In the case of Yoruba (YRI) as a hybrid between Gambian Mandinka (GWD) and Luhya (LWK) the accuracy of the post-JL angle is extremely poor (8.3%). In fact, the resulting post-JL angle is further away from the ideal 180° mark than the pre-JL angle. Another made-up scenario of Britons (GBR) as mixture of Spaniards (IBS) and Finns (FIN) has a pre-JL angle smaller that 90°, with a poor post-JL angle reconstruction (68.3%).

The two last entries in Table S.1 are instances of whimsically chosen populations. Although the admixture proportions are in range, the pre-JL and post-JL indices in Table S.1 point out that these models are flatly rejected.

We have observed that whereas in cases of successful admixture models the slope estimates with the two sets of auxiliary populations are very similar, unsuccessful models tend to give differences in slopes larger that the confidence intervals provided by the fitting routine.

Three-way admixture examples with real data are provided in Table S.2. When estimating Colombians (CLM) and Puerto Ricans (PUR) as a mix of three known sources present in modern Latin American populations we obtain valid admix-

ture models. In both cases, the flatness index value decreases by one order of magnitude after JL projection. On the other hand, the two three-way made-up examples with Khomani and Papuan as potential hybrids provide inconsistent values for the coefficients in one case and no significant decreasing for the post-JL flatness index (Table S.2).

S.14 Two-step three-way admixture

Let us assume that (S.27) has been resolved for α and β and consider a variant of the problem where we can suppose that the underlying history of the admixture is actually a two-step process. We know the first two contributors, say a and b (with unknown proportion μ), which give rise to a transitional hybrid z. A time later, z gets admixed with a third parental c (with unknown proportion ν). The LS solutions α and β allows us to estimate the mixture proportions, μ, ν, in each admixture phase by considering the two linear combinations

$$\begin{aligned} \vec{p}_z &= \mu\vec{p}_a + (1-\mu)\vec{p}_b \\ \vec{p}_x &= \nu\vec{p}_c + (1-\nu)\vec{p}_z. \end{aligned} \tag{S.31}$$

The first one describes the admixture of populations a, b with proportion μ, and the second one the admixture of c with the transitional z, with proportion ν. When combined

$$\vec{p}_x = (1-\nu)\mu\vec{p}_a + (1-\nu)(1-\mu)\vec{p}_b + \nu\vec{p}_c. \tag{S.32}$$

Comparison of the coefficients of the linear combinations (10) and (S.32) yields the 2-step-3-way admixture proportions

$$\mu = \frac{\alpha}{\alpha+\beta}, \quad \nu = 1-\alpha-\beta. \tag{S.33}$$

Due to the error propagation rules, the determinations μ and ν are less accurate than those of α, β, γ. This is natural consequence that the SNP content of the transitional hybrid, a piece of the problem, is unknown.

S.15 Discrete model of gene flow

The gene flow is a great drawback in the admixture problem analysis. Next we propose a modest approach that addresses the issue as sequence of n 2-way processes. We pose two different scenarios.

Let us consider a variant of the problem in which repeated 2-way admixtures occur with donor b giving rise to successive transitional hybrid populations denoted by vector $\vec{p}_k$, with $k = 1, 2, \ldots, n$

$$\begin{aligned} \vec{p}_1 &= \alpha_1\vec{p}_a + (1-\alpha_1)\vec{p}_b, \\ \vec{p}_2 &= \alpha_2\vec{p}_1 + (1-\alpha_2)\vec{p}_b, \\ &\vdots \\ \vec{p}_n &= \alpha_n\vec{p}_{n-1} + (1-\alpha_n)\vec{p}_b. \end{aligned} \tag{S.34}$$

Now, if we consider the problem in one step

$$\vec{p}_n = \alpha\vec{p}_a + (1-\alpha)\vec{p}_b, \tag{S.35}$$

we can conclude, after a little algebra,

$$\alpha = \alpha_1\alpha_2\ldots\alpha_n, \tag{S.36}$$

and hence, for n moderately large, $\alpha \to 0$, due to the fact $\alpha_k < 1$. This scenario is, so to speak, a *multiplicative route* to introgression. None of the intermediate admixture steps is an introgression regime but the final scenario is. Figure S.9a gives a planar scheme of a possible realization of this phenomenon in the allele frequency space. Repeated admixture of the transitional hybrid (red dots) with donor b (gray dot) makes the hybrid to approach b after five steps although none of the mixture processes is in the introgression regime. The displacements induced by genetic drift on the admixture triangles are represented in Figure S.9b.

A more general gene flow scenario takes place whenever in each 2-way admixture process a new donor is introduced. It is required the knowledge of the set of contributors $\{c_k\}$, $k = 0, \ldots, n$, as well as their ordering. We get then the system

$$\begin{aligned} \vec{p}_1 &= \alpha_1\vec{c}_0 + (1-\alpha_1)\vec{c}_1, \\ \vec{p}_2 &= \alpha_2\vec{p}_1 + (1-\alpha_2)\vec{c}_2, \\ &\vdots \\ \vec{p}_n &= \alpha_n\vec{p}_{n-1} + (1-\alpha_n)\vec{c}_n, \end{aligned} \tag{S.37}$$

where $p_k, k = 1, \ldots, n-1$, are transitional hybrids and p_n is the hybrid population proxy, so that

$$\vec{p}_n = \mu_0\vec{c}_0 + \ldots + \mu_n\vec{c}_n, \tag{S.38}$$

with the mixing coefficients μ's. We assume that we have $\{\mu_k\}$ estimates via a generalization of the LS procedure introduced in 2- and 3-way admixture exposed above. It is not difficult to express the α's in terms of the estimated μ's

$$\begin{aligned} \alpha_1 &= \frac{\mu_0}{\mu_0+\mu_1}, \\ \alpha_2 &= \frac{\mu_0+\mu_1}{\mu_0+\mu_1+\mu_2}, \\ &\vdots \\ \alpha_{n-1} &= \frac{\mu_0+\mu_1+\ldots+\mu_{n-2}}{\mu_0+\mu_1+\mu_2+\ldots+\mu_{n-1}}, \\ \alpha_n &= \mu_0+\ldots+\mu_{n-1} = 1-\mu_n. \end{aligned} \tag{S.39}$$

These relationships are such that if all the μ's are in range then the α's are as well.

Whenever one μ value dominates in (S.39), say $\mu_m \to 1$, and concomitantly $\mu_k \ll 1$, $\forall k \neq m$, some conclusions can be drawn. The first proportions, $\alpha_1, \ldots, \alpha_{m-1}$, may get any value; the coefficient $\alpha_m \to 0$, and the remaining ones $\alpha_{m+1}, \ldots, \alpha_n \to 1$. The interpretation is that at the step m the donor c_m dominates over the transitional p_{m-1} in (S.39). Thus, the previous admixture processes could be approximately ignored. The subsequent α's tend to unit, which witnesses introgression processes so that p_m and p_n differs very little.

Under the less restrictive situation $\mu_m \to 1$ and $\mu_k \ll 1$, $\forall k < m$, the conclusion is that the $m-1$ previous admixture processes can be dropped out in the analysis, without any further ado.

S.16 Tetrahedron and flatness index

Some features of the tetrahedron in terms of edge lengths are gathered. Let V be the tetrahedron volume, A the base area, and h the height (determined by the hybrid vertex). We have $V = Ah/3$.

The length of edge $\overline{ij}$ is d_{ij} and we assume that the hybrid is located at vertex 1 The volumen may be calculated using the six edge lengths with the Cayley-Menger determinant [12, 10]

$$288V^2 = \begin{vmatrix} 0 & 1 & 1 & 1 & 1 \\ 1 & 0 & d_{12}^2 & d_{13}^2 & d_{14}^2 \\ 1 & d_{12}^2 & 0 & d_{23}^2 & d_{24}^2 \\ 1 & d_{13}^2 & d_{23}^2 & 0 & d_{34}^2 \\ 1 & d_{14}^2 & d_{24}^2 & d_{34}^2 & 0 \end{vmatrix}. \quad \text{(S.40)}$$

In the allele frequency space, $d_{ab}^2 = (\vec{p}_a - \vec{p}_b)^2 = s\, f_2(a,b)$. In the JL subspace, $d_{ab}^2 = (\vec{p}_a - \vec{p}_b) \cdot (\vec{p}_i - \vec{p}_j)/|\vec{p}_i - \vec{p}_j| = s \sum_{k<l} [f_4'(a,b;k,l)]^2$. Explicitly

$$\begin{aligned} 144V^2 = &- d_{12}^4\, d_{34}^2 - d_{12}^2\, d_{13}^2\, d_{23}^2 + d_{12}^2\, d_{13}^2\, d_{24}^2 + d_{12}^2\, d_{13}^2\, d_{34}^2 \\ &+ d_{12}^2\, d_{14}^2\, d_{23}^2 - d_{12}^2\, d_{14}^2\, d_{24}^2 + d_{12}^2\, d_{14}^2\, d_{34}^2 \\ &+ d_{12}^2\, d_{23}^2\, d_{34}^2 + d_{12}^2\, d_{24}^2\, d_{34}^2 - d_{12}^2\, d_{34}^4 - d_{13}^4\, d_{24}^2 \\ &+ d_{13}^2\, d_{14}^2\, d_{23}^2 + d_{13}^2\, d_{14}^2\, d_{24}^2 - d_{13}^2\, d_{14}^2\, d_{34}^2 + d_{13}^2\, d_{23}^2\, d_{24}^2 \\ &- d_{13}^2\, d_{24}^4 + d_{24}^2\, d_{13}^2\, d_{34}^2 - d_{14}^4 d_{23}^2 - d_{14}^2\, d_{23}^4 \\ &+ d_{14}^2\, d_{23}^2\, d_{24}^2 + d_{23}^2\, d_{14}^2\, d_{34}^2 - d_{23}^2\, d_{24}^2\, d_{34}^2. \end{aligned} \quad \text{(S.41)}$$

The base area may be computed with the Heron formula [10], which involves the three triangle edges

$$\begin{aligned} A^2 &= p(p - d_{23})(p - d_{24})(p - d_{34}) \\ 2p &= (d_{23} + d_{24} + d_{34}). \end{aligned} \quad \text{(S.42)}$$

Combining those results we get the tetrahedron height, $h = 3V/A$, in terms of its edge lengths and define a dimensionless flatness index as the ratio $h/\sqrt{A}$. In terms of volume and base area it reads: $3V/A^{3/2}$, to be computed with (S.41) and (S.42).

The Cayley-Menger determinant (S.40) can be used to show explicitly that in a 3-way admixture at its inception the four populations form a co-planar configuration. It suffices to use the linear combination $\vec{p}_1 = \alpha\vec{p}_2 + \beta\vec{p}_3 + (1-\alpha-\beta)\vec{p}_4$, to compute the distances $d_{1,k}$, with $k = 2, 3, 4$, in (S.40). The result of the determinant factorizes as $(d_{23}^2 - d_{24}^2 - d_{34}^2 + 2d_{24}d_{34}C) \cdot Q$, where Q is a factor that is irrelevant for our goal and, more crucially, C is the cosine of the angle between edges $\overline{24}$ and $\overline{34}$. The determinant vanishes because the first factor does as a result of the cosine law applied to $\triangle 234$.

The general geometric interpretation of the mixing coefficients in the 3-way case, (S.28) and (S.30), requires a number of projections in 3D that renders lengthy the explanation. However, at the inception of population admixture, when the four point structure is planar, the situation may be easily illustrated. The various orthogonal projections involved in the scalar products are visualized in Figure S.10. The three donors in the allele frequency space form the triangle $\triangle abc$ with the hybrid inside. Capital letters stand for segment lengths. After some algebra, it is straightforward to write down the coefficients (S.30) at the inception of admixture in terms of lengths and angles

$$\alpha = \frac{Z}{B}\frac{\sin\theta'}{\sin\varphi} = \frac{P}{Q}, \quad \beta = \frac{Z}{A}\frac{\sin\theta}{\sin\varphi} = \frac{U}{V}. \quad \text{(S.43)}$$

References

[1] 1000 Genomes Consortium. A global reference for human genetic variation. *Nature 2015 526:7571*, 526:68–74, 9 2015.

[2] L. L. Cavalli-Sforza, P. Menozzi, and A. Piazza. *The history and geography of human genes*. Princeton University Press, 1994.

[3] David Reich Lab. Github-DReichLab/AdmixTools: Tools test whether admixture occurred and more, 2023. https://github.com/DReichLab/AdmixTools.

[4] Wolfgang Haak et al. Massive migration from the steppe was a source for Indo-European languages in Europe. *Nature*, 522:207–211, 2015.

[5] Eadaoin Harney, Nick Patterson, David Reich, and John Wakeley. Assessing the performance of qpAdm: A statistical tool for studying population admixture. *Genetics*, 217:iyaa045, 2021.

[6] Iosif Lazaridis et al. Genomic insights into the origin of farming in the ancient Near East. *Nature*, 536:419–424, 2016.

[7] José-Angel Oteo and Gonzalo Oteo-García. Mutations along human chromosomes: How randomly scattered are they? *Physical Review E*, 106:064404, 2022.

[8] Gonzalo Oteo-García and José-Angel Oteo. A Geometrical Framework for f-Statistics. *Bulletin of Mathematical Biology*, 83:14, 2021.

[9] Nick Patterson, Priya Moorjani, Yontao Luo, Swapan Mallick, Nadin Rohland, Yiping Zhan, Teri Genschoreck, Teresa Webster, and David Reich. Ancient admixture in human history. *Genetics*, 192:1065–93, 2012.

[10] Duncan M. Y. Sommerville. *Introduction to the Geometry of N Dimensions*. Courier Dover Publications, 2020.

[11] Roman Vershynin. *High-Dimensional Probability: An Introduction with Applications in Data Science*. Cambridge University Press, 2018.

[12] Karl Wirth and André S. Dreiding. Edge lengths determining tetrahedrons. *Elemente der Mathematik*, 64:160–170, 2009.

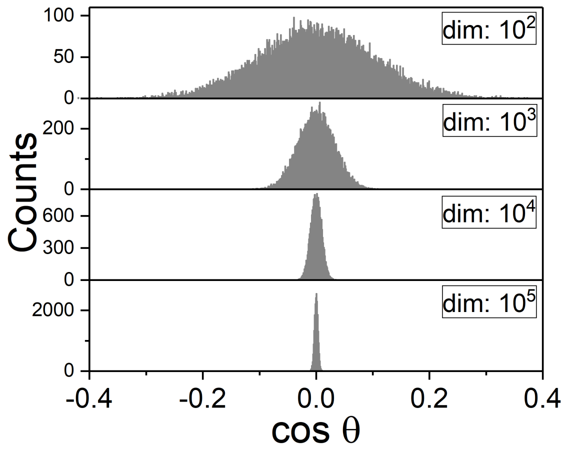

Figure S.1: Quasi-orthogonality. Histograms of $\cos\theta = \vec{u}\cdot\vec{z}/(|\vec{u}||\vec{z}|)$, with $\vec{u} = (1, \ldots, 1)$, evaluated in four different dimensions. The distribution gets narrower as the space dimension increases.

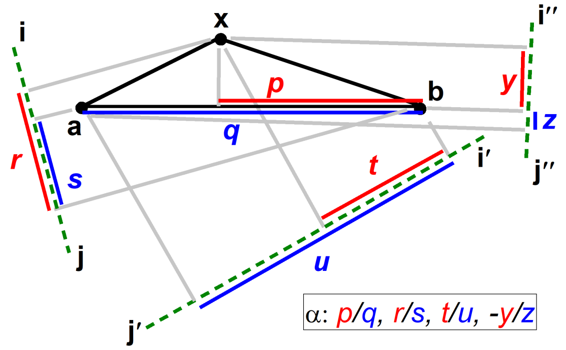

Figure S.2: Planar representation for different estimates of the f_4-ratio. Symbols p, q, r, s, t, u, y, z, stand for segment lengths. The ratio p/q is from (S.6), and r/s, t/u, $-y/z$ are the f_4-ratio (8) evaluated with three different pairs of auxiliary populations. Triangle $\triangle axb$ is from Figure 1. Dashed green lines are directions defined by auxiliary pairs. Gray lines are eye guides for the orthogonal projections of the edges of the triangle. Red and blue segments are projections of the triangle sides $\overline{xb}$ and $\overline{ab}$, respectively.

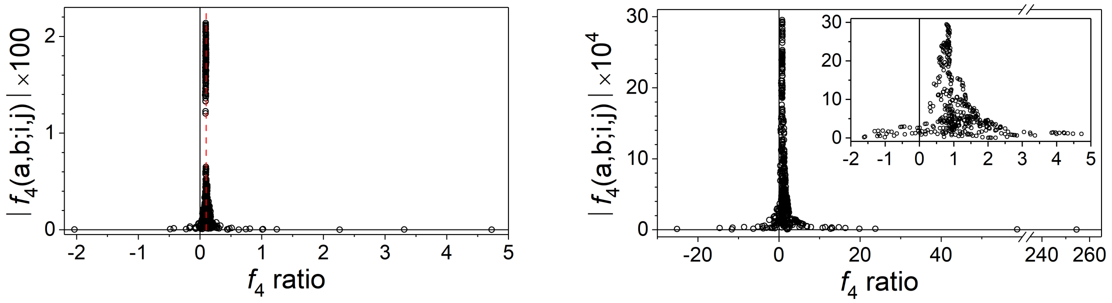

(a) Simulated: 0.1·English+0.9·Yoruba

(b) Real data: Italian_North = α·Basque $+(1-\alpha)$·Adygei

Figure S.3: Behaviour of the f_4-ratio values with respect to the shared drift. Panel (a) is for the simulated case 0.1·English+0.9·Yoruba, and $\epsilon = 0.5$. The dashed line stands for the nominal $\alpha = 0.1$. Panel (b) is for the unlikely real data model: Italian_North $= \alpha$·Basque $+(1-\alpha)$·Adygei. The zoomed area in the inset has the same horizontal scale as panel (a).

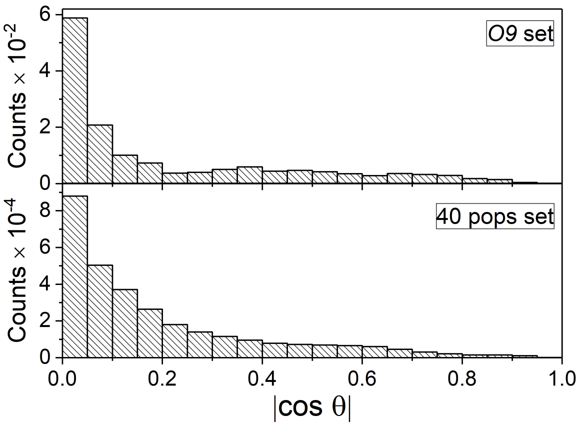

Figure S.4: Quasi-orthogonality in allele frequency space. Histogram of the absolute values of the cosine of the angle θ between two auxiliary pairs. Top and bottom panels are, respectively, for the **O9** and the 40 populations dataset.

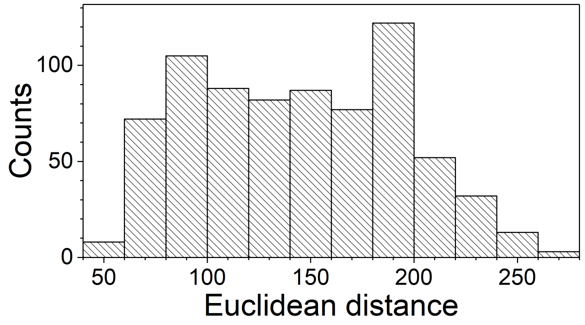

Figure S.5: Histogram of Euclidean distances ($= \sqrt{s\, f_2}$) provided by auxiliary population pairs in the dataset.

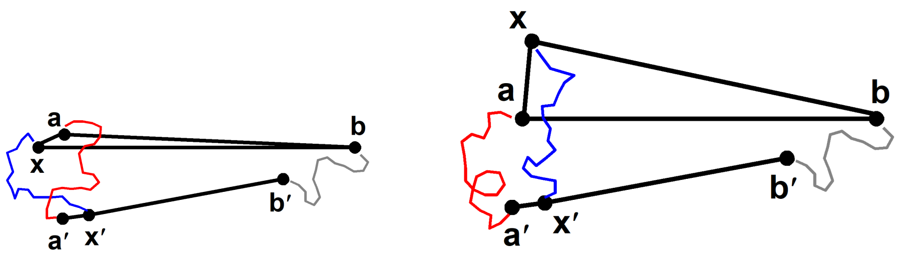

(a) Exchange of donor-hybrid roles: the x vertex angle becomes acute.

(b) Admixture becomes phylogeny: all angles are acute.

Figure S.6: Planar representation of two introgression instances where genetic drift leads to misleading configurations in allele frequency space. Triangle $\triangle axb$ and segment $\overline{a'x'b'}$ are as in Figure 1.

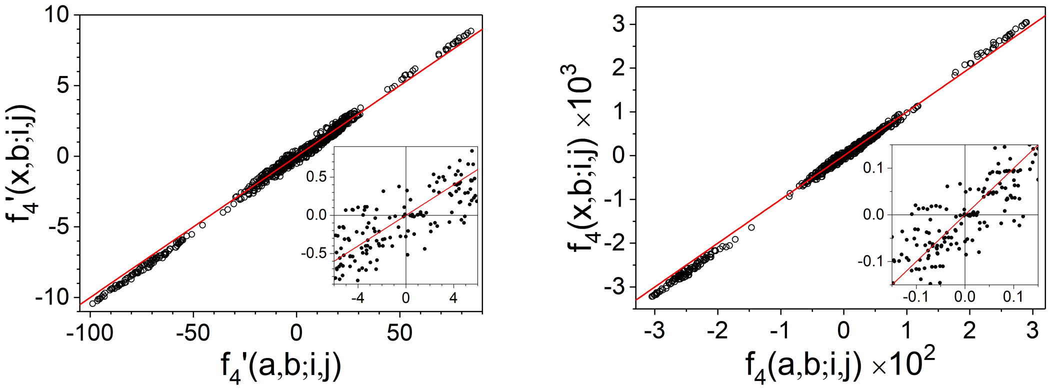

(a) Renormalized (b) Standard

Figure S.7: 2D representations of population admixture. Simulated model: 0.1·English + 0.9·Yoruba, with noise amplitude $\epsilon = 0.5$. Panel (a) is for the renormalized representation (S.10) and panel (b) is for the standard representation (S.22). Solid lines have slope 0.1. The zoomed area allows to appreciate the diversity of slopes one can get in an f_4-ratio evaluation where the denominator is small by considering the slope defined by every single point.

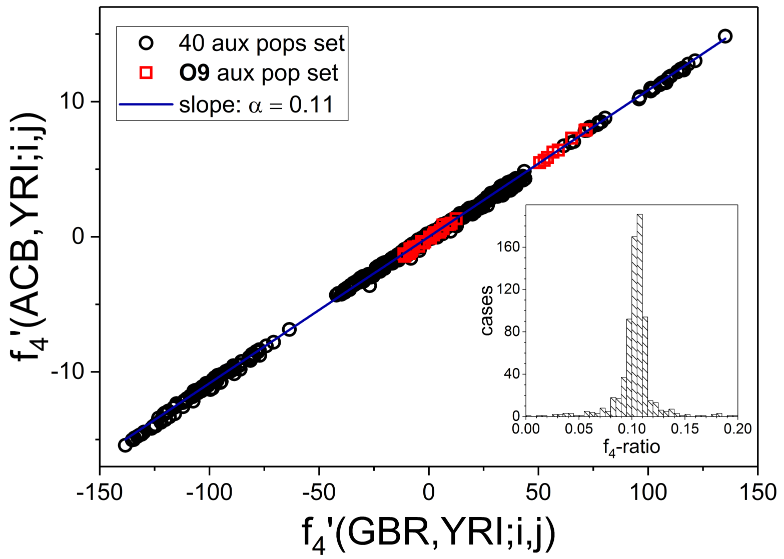

Figure S.8: Real data population admixture model ACB.SG=α·GBR.SG+$(1-\alpha)$·YRI.SG. Solid line is the linear fit through the origin whose slope is the α estimate. Black circles and red squares stand for the 40 auxiliary population set and the O9 set, respectively. The number of SNPs is different in both sets. The two slope estimates are the same. The inset is a histogram of f_4-ratio values, with mean, 0.11, and standard deviation, 0.05, obtained with the values that fall within the range $[0, 1]$.

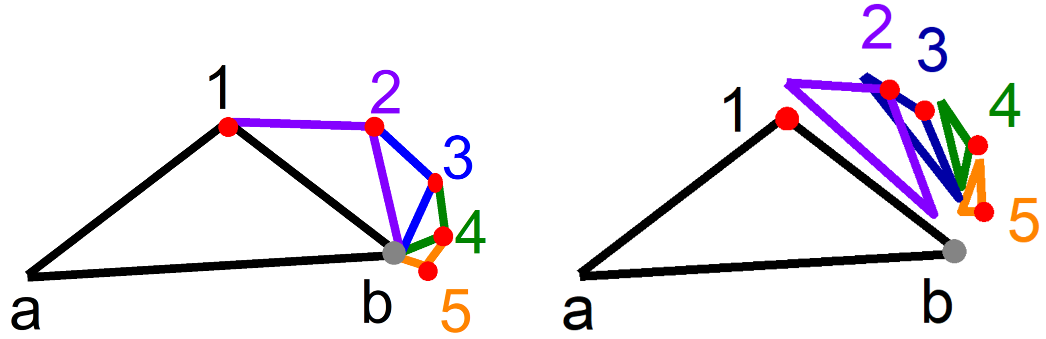

(a) (b)

Figure S.9: Multiplicative route to introgression from donors a and b in allele frequency space. (a) Repeated admixture of the transitional hybrid (red dot) with donor b (gray dot) makes the hybrid to approach b after five steps. None of the mixture processes is in the introgression regime. (b) The displacements induced by genetic drift on the admixture triangles have been represented here.

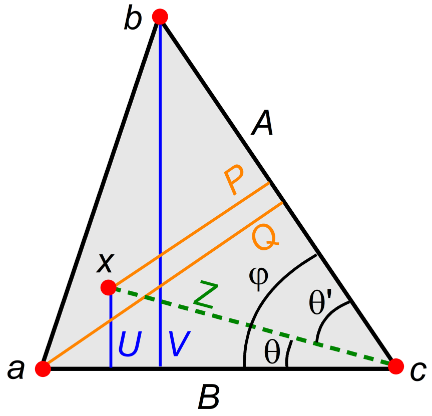

Figure S.10: Planar structure in allele frequency space of the 3-way population admixture at inception. Interpretation of the mixing coefficients: $\alpha = P/Q$ and $\beta = U/V$, where (symbolically) $x = \alpha \cdot a + \beta \cdot b + (1 - \alpha - \beta) \cdot c$. Capital letters stand for lengths.

Table S.1: Two-way admixture models with real data. Besides the admixture proportions, the values of the admixture f_3-test, the pre-Jl and post-JL angles are given. Remind that 180° is an ideal mixture. The last two columns stand for the relative angle in percentage.

				Angle (deg)		Angle/180 (%)	
Model	α	β	f_3-test	pre-JL	post-JL	pre-JL	post-JL
ACB=α·GBR+β·YRI	0.11	0.89	−0.0031	106	174	58.9	96.7
MXL=α·IBS+β·PEL	0.36	0.64	−0.0026	106	173	58.9	96.1
YRI=α·LWK+β·GWD	−1.24	2.24	0.0015	68	15	37.8	8.3
GBR=α·TSI+β·FIN	0.67	0.33	0.0012	72	123	40.0	68.3
Sardinian=α·Italian_North+β·Basque	0.98	0.02	0.0035	59	47	33	26
Italian_North=α·Basque+β·Adygei	0.80	0.20	0.0025	69	116	38	64

Table S.2: Three-way admixture models with real data. Admixture coefficients and pre-JL and post-JL flatness indexes.

				Flatness	
Model	α	β	γ	pre-JL	post-JL
CLM=α·IBS+β·YRI+γ·PEL	0.58	0.07	0.36	0.387	0.008
PUR=α·IBS+β·GWD+γ·PEL	0.68	0.13	0.19	0.386	0.011
Khomani=α·Hazara+β·Aleut+γ·Mayan	1.61	−0.11	−0.49	0.778	0.325
Papuan=α·Mayan+β·Khomani+γ·Saami	0.46	0.20	0.34	1.263	0.710
